# Supplementary material for: Enhancing radiation-resistance and peroxidase-like activity of single-atom copper nanozyme via local coordination manipulation
Source: Nat Commun. 2024 Jul 22;15:6174. doi: 10.1038/s41467-024-50416-8 (PMC11263674; doi:10.1038/s41467-024-50416-8)
Supplement: Supplementary file 1 — Supplementary Information [file 41467_2024_50416_MOESM1_ESM.pdf]

Supplementary Information

Enhancing Radiation-resistance and Peroxidase-like Activity of Single-atom Copper Nanozyme via Local Coordination Manipulation

Jiabin Wu[†1], Xianyu Zhu[†2,3], Qun Li[4], Qiang Fu[*5,6], Bingxue Wang[5], Beibei Li[1], Shanshan Wang[7], Qingchao Chang[2], Huandong Xiang[2,8], Chengliang Ye[1], Qiqiang Li[3], Liang Huang[4], Liang Yan[*2], Dingsheng Wang[1], Yuliang Zhao[2,8], Yadong Li[*1,9,10]

[1]Department of Chemistry, Tsinghua University, Beijing 100084, China

[2]CAS Key Laboratory for Biomedical Effects of Nanomaterials and Nanosafety, Institute of High Energy Physics and National Center for Nanoscience and Technology, Chinese Academy of Sciences, Beijing 100049, China

[3]Institute of Marine Science and Technology, Shandong University, Qingdao 266237, China

[4]Wuhan National Laboratory for Optoelectronics, Huazhong University of Science and Technology, Wuhan 430074, China

[5]School of Future Technology, University of Science and Technology of China, Hefei 230026, China

[6]Hefei National Laboratory, University of Science and Technology of China, Hefei 230088, China

[7]Institute of Quality Standards & Testing Technology for Agro-Products, Chinese Academy of Agricultural Sciences, Beijing 100081, China

[8]GBA Research Innovation Institute for Nanotechnology, Guangdong 510700, China

[9]College of Chemistry, Beijing Normal University, Beijing 100875, China

[10]The Key Laboratory of Functional Molecular Solids, Ministry of Education, College of Chemistry and Materials Science, Anhui Normal University, Wuhu 241002, China

[†]These authors contributed equally: Jiabin Wu and Xianyu Zhu

Emails: qfu3@ustc.edu.cn; yanliang@ihep.ac.cn; ydli@mail.tsinghua.edu.cn

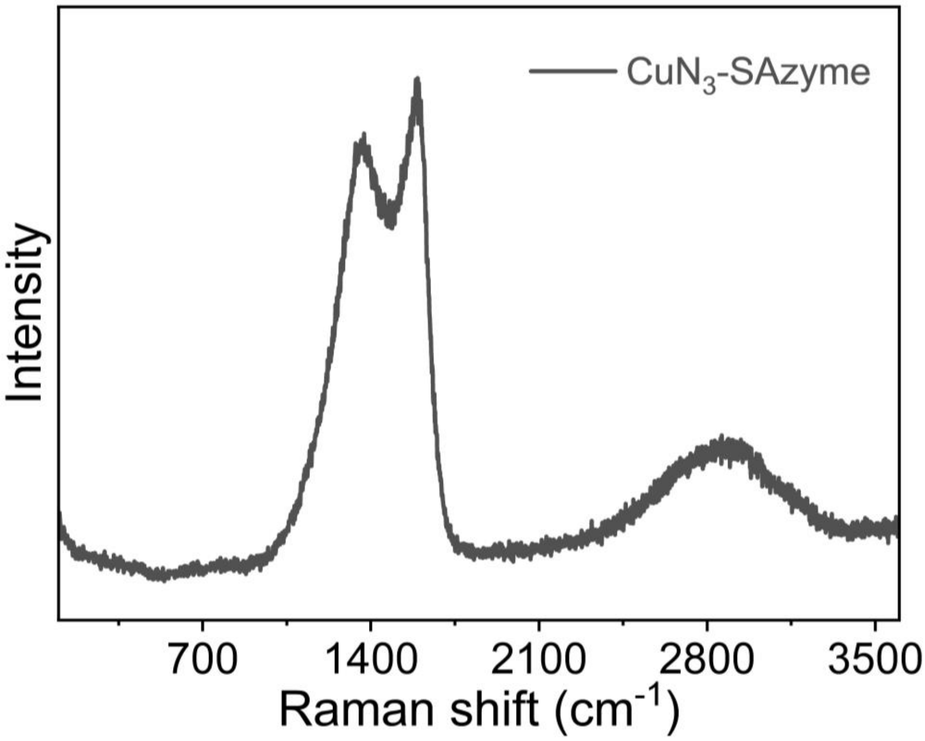

Supplementary Figure 1. Raman spectrum of CuN_3-SAzyme.

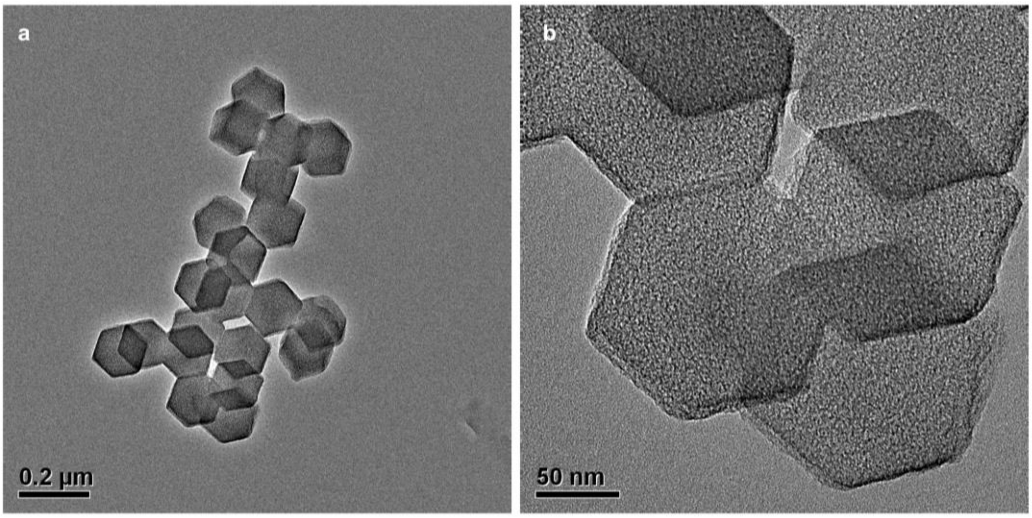

Supplementary Figure 2. TEM (**a**) and HR-TEM (**b**) images of CuN_4-SAzyme. Three times each morphology characterization was repeated independently with similar results. Representative images are presented.

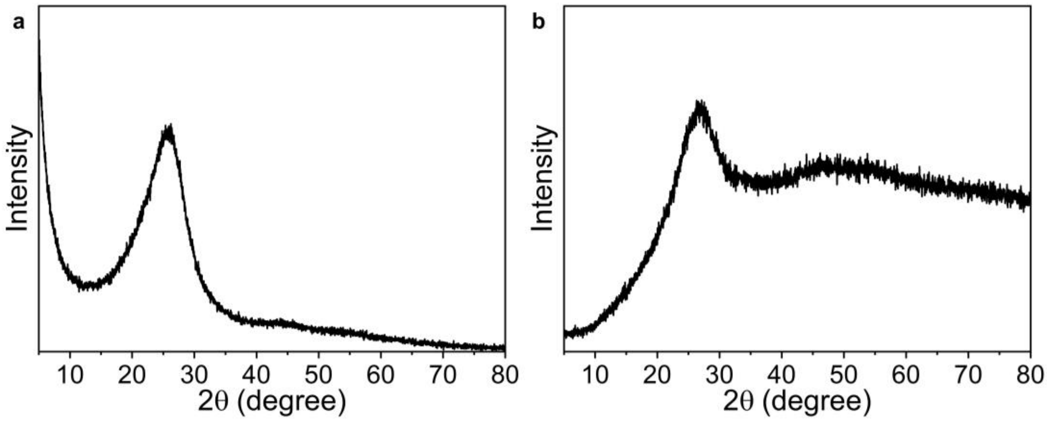

Supplementary Figure 3. XRD patterns of CuN_3-SAzyme (**a**) and CuN_4-SAzyme (**b**).

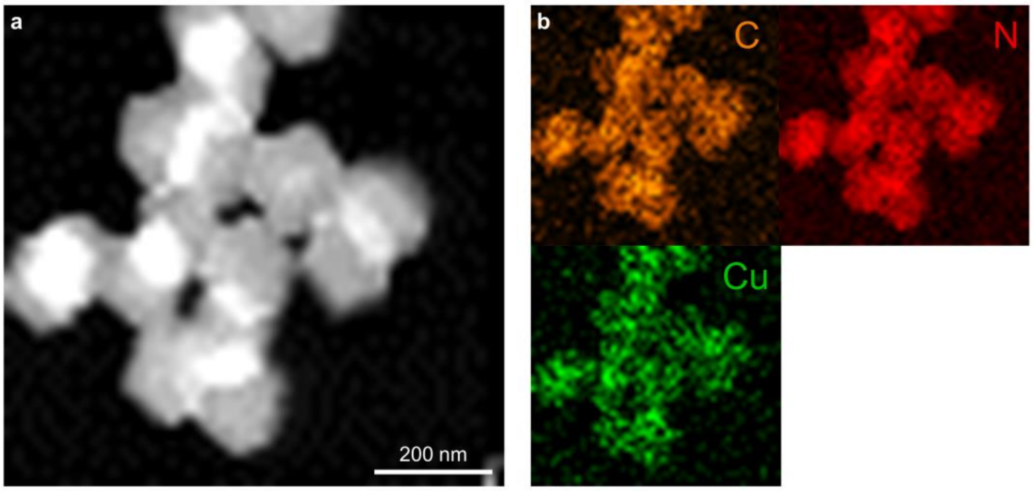

Supplementary Figure 4. HAADF-STEM image (**a**) and corresponding EDS mapping (**b**) of CuN_4-SAzyme. Three times each morphology characterization was repeated independently with similar results. Representative images are presented.

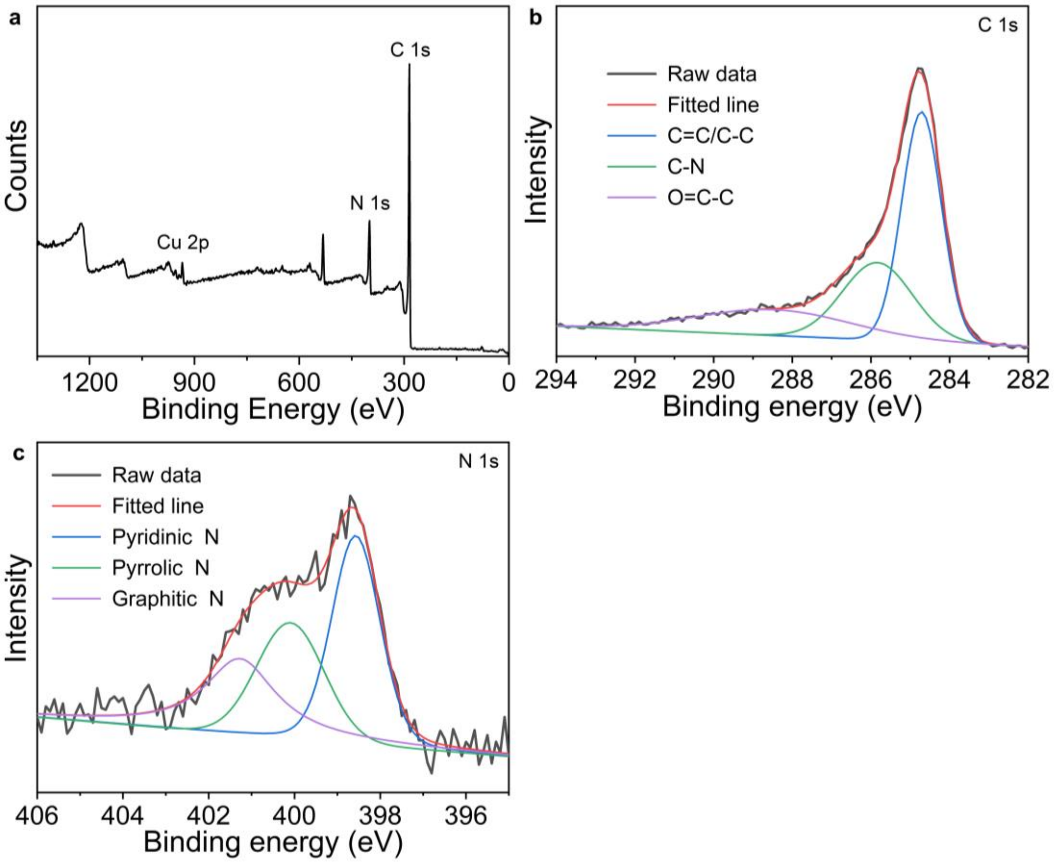

Supplementary Figure 5. XPS analysis of CuN_3-SAzyme. a XPS survey spectrum. **b-c** C 1s (**b**) and N 1s (**c**) XPS spectra.

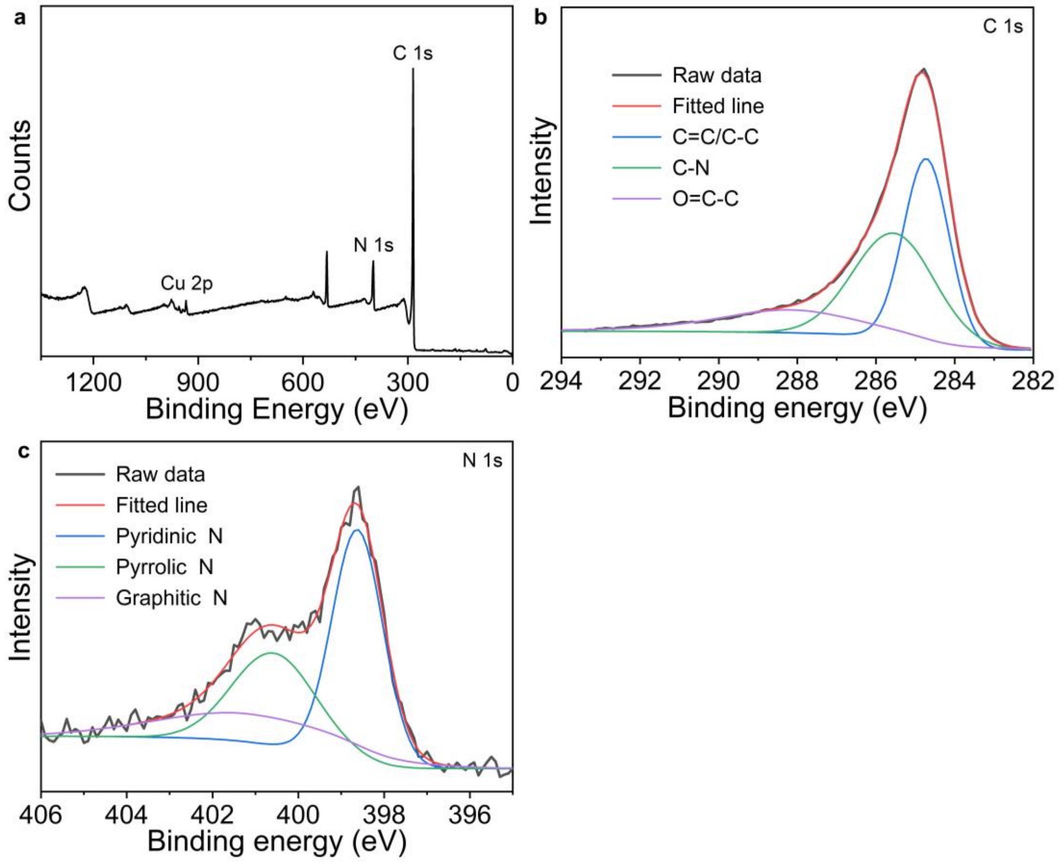

Supplementary Figure 6. XPS analysis of CuN_4-SAzyme. a XPS survey spectrum. **b-c** C 1s (**b**) and N 1s (**c**) XPS spectra.

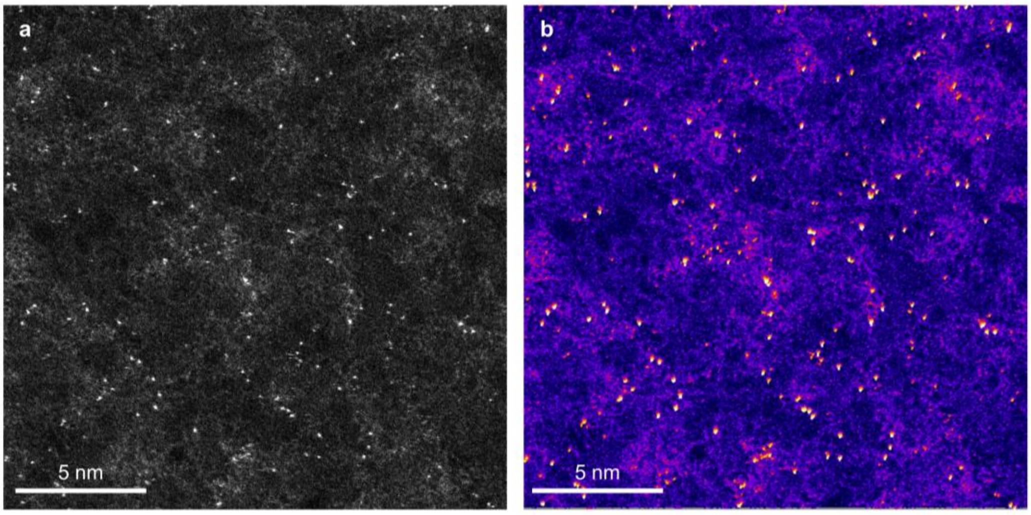

Supplementary Figure 7. AC HAADF-STEM image (**a**) and Corresponding surface intensity map (**b**) of CuN_4-SAzyme. Three times each morphology characterization was repeated independently with similar results. Representative images are presented.

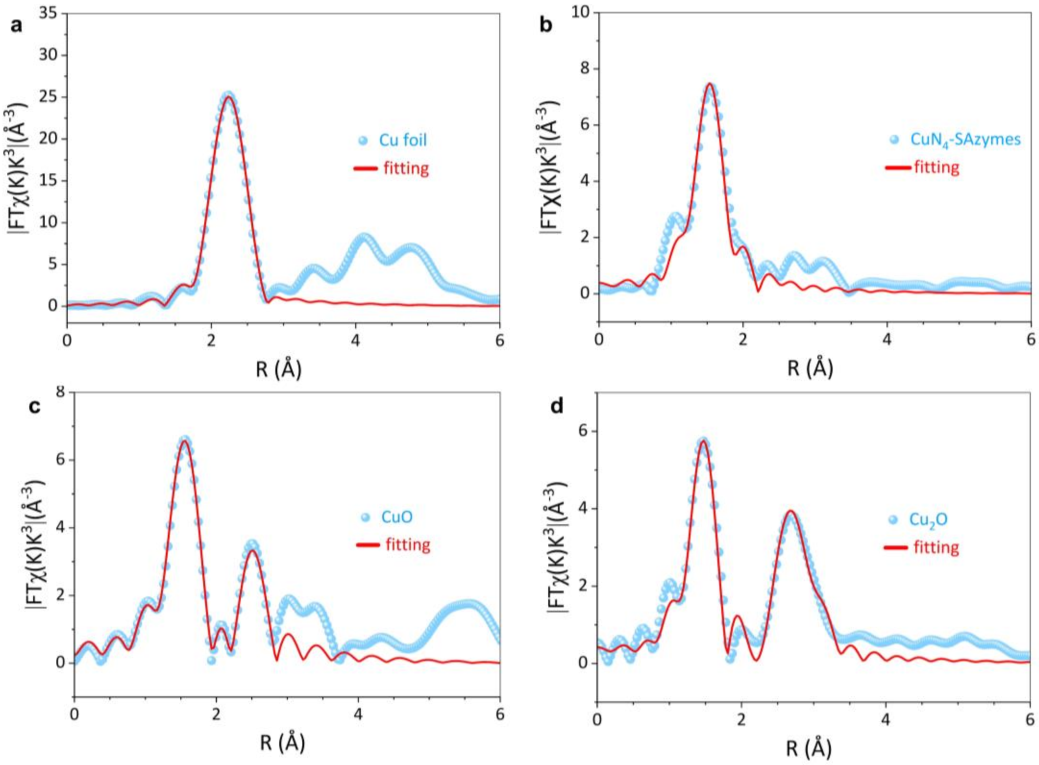

Supplementary Figure 8. *R*-space FT-EXAFS fitting plots of Cu foil (**a**), CuN_4-SAzyme (**b**), CuO nanozyme (**c**), and Cu_2O nanozyme (**d**).

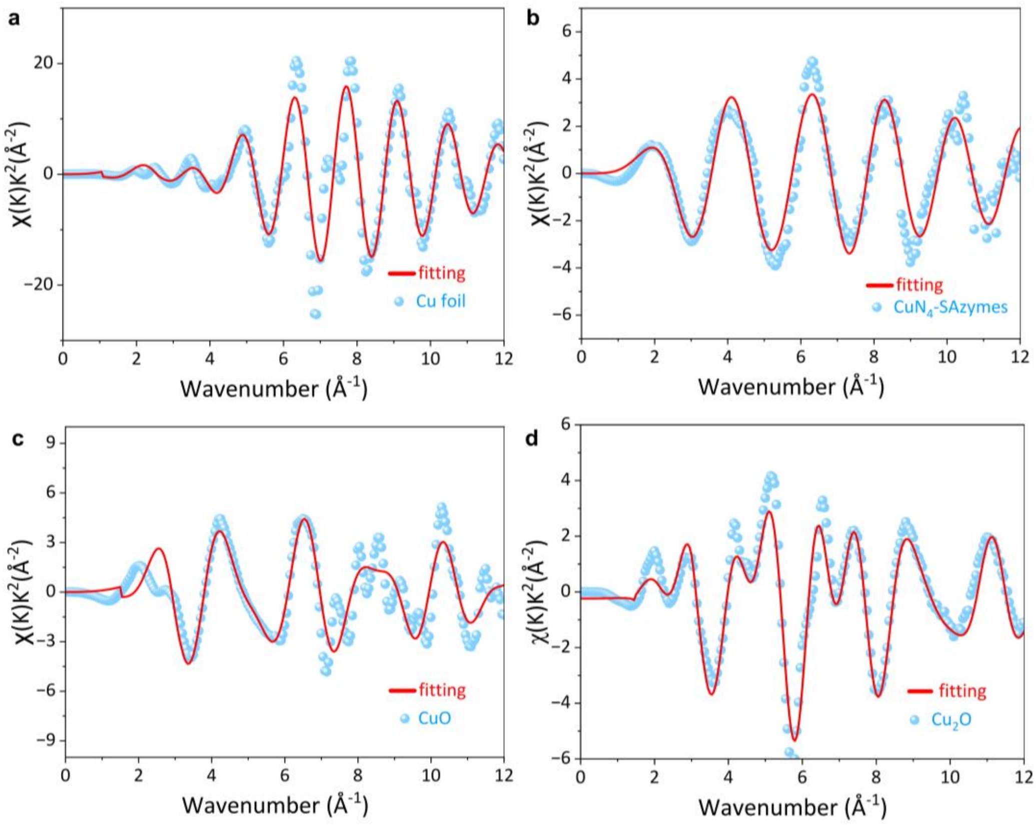

Supplementary Figure 9. *k*-space FT-EXAFS fitting plots of Cu foil (**a**), CuN_4-SAzyme (**b**), CuO nanozyme (**c**), and Cu_2O nanozyme (**d**).

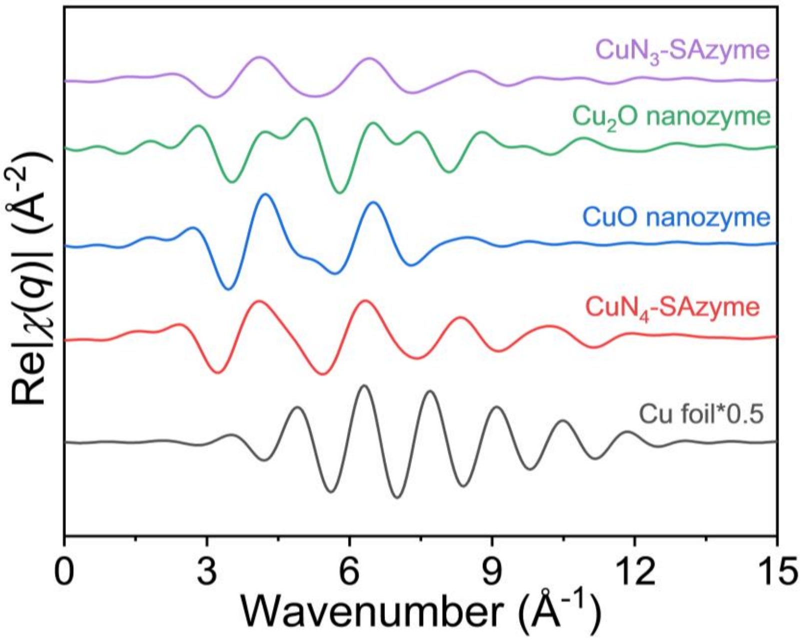

Supplementary Figure 10. q-space FT-EXAFS plots of CuN_x-SAzymes, CuO_x nanozymes, and Cu foil.

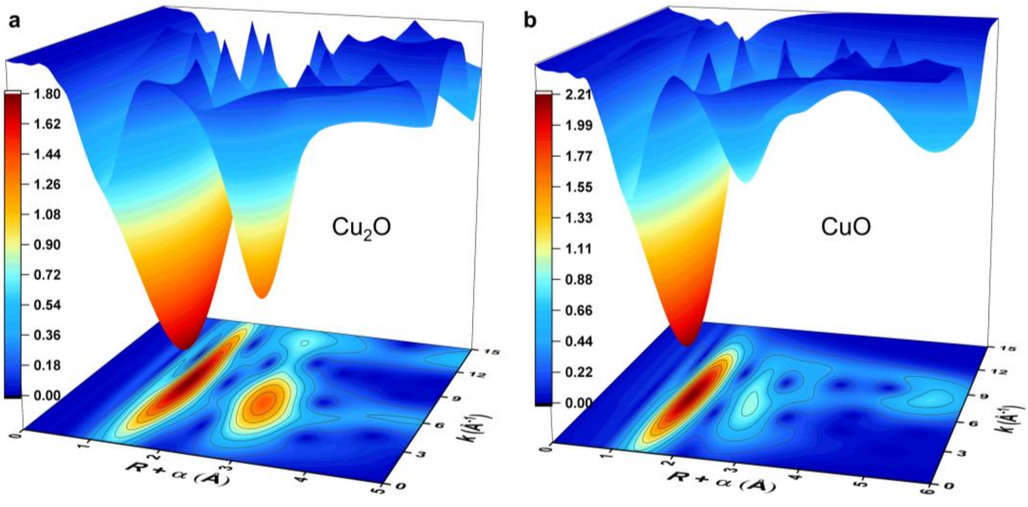

Supplementary Figure 11. WT curves of Cu_2O nanozyme (**a**) and CuO nanozyme (**b**).

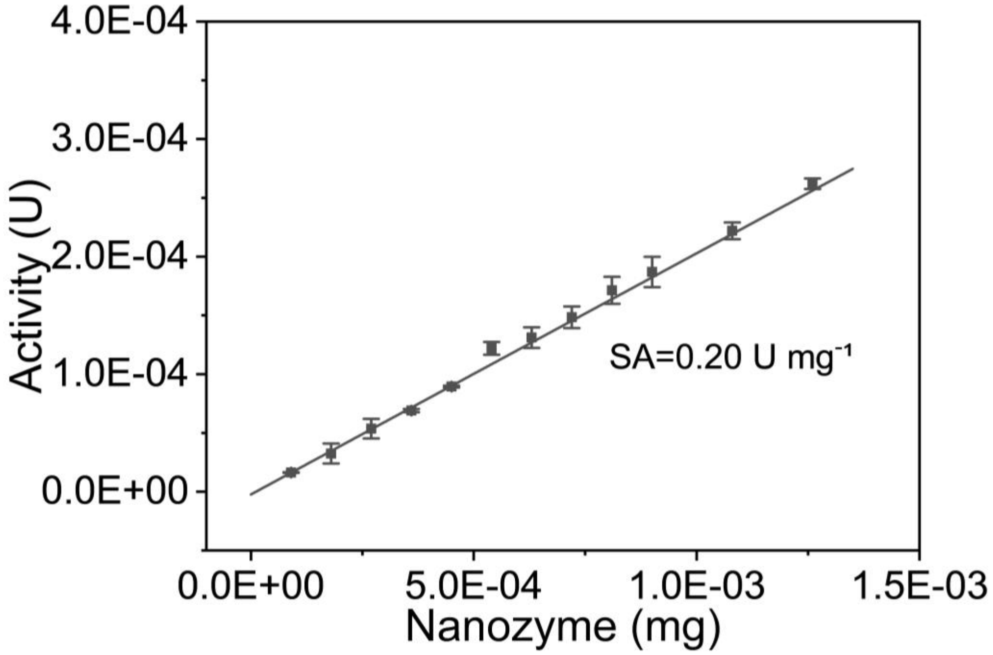

Supplementary Figure 12. Enzymatic activity of Cu-free N-doped carbon support. These data are presented as mean values ± SD (n = 3 independent experiments).

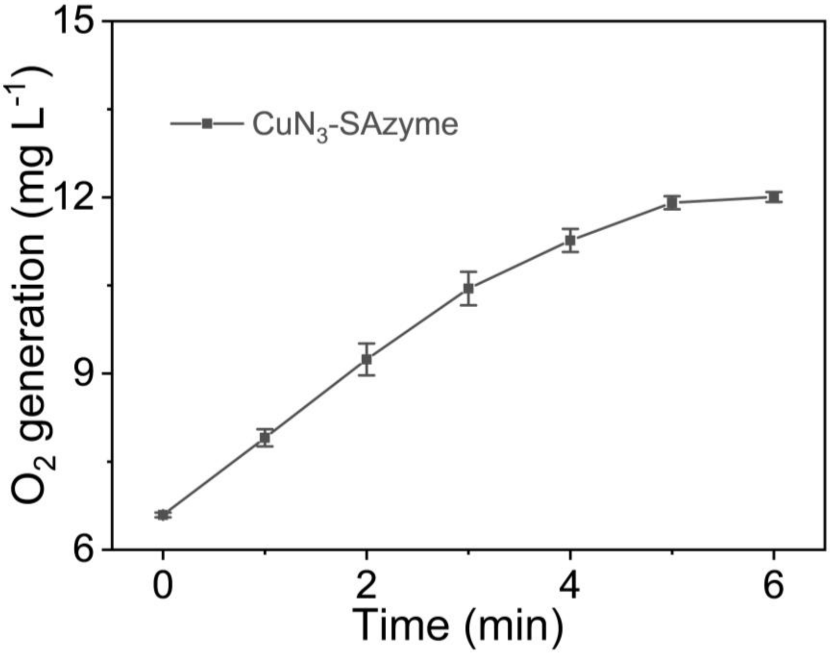

Supplementary Figure 13. The catalase-like activity of CuN_3-SAzyme. These data are presented as mean values ± SD (n = 3 independent experiments).

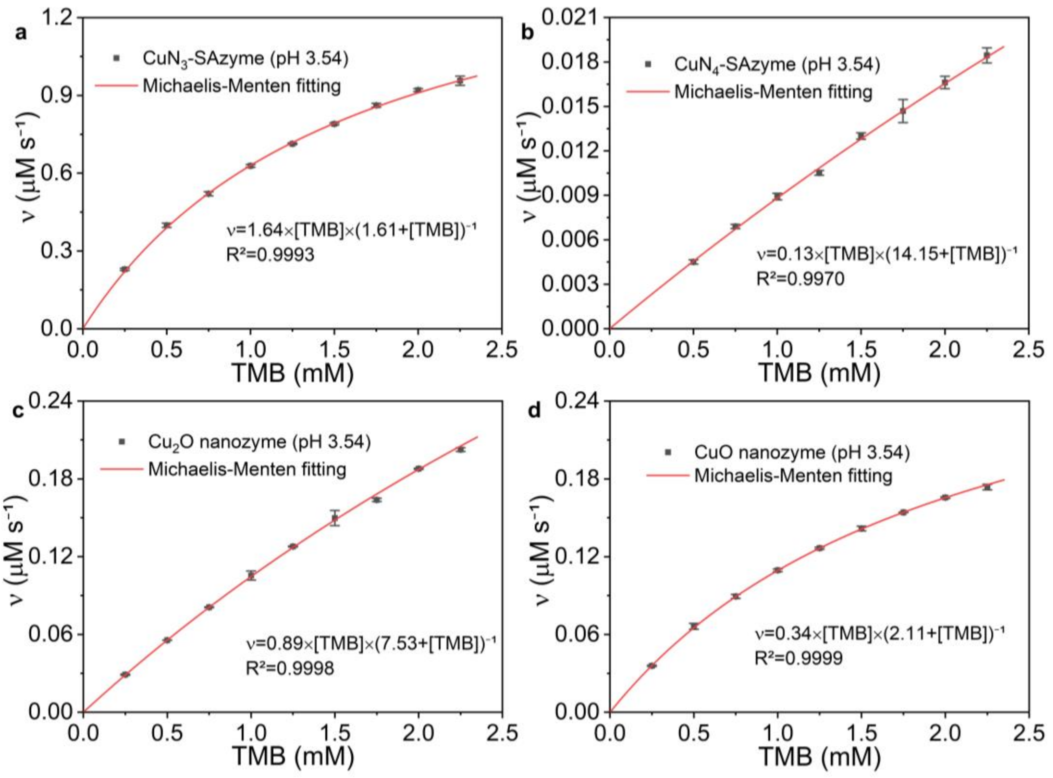

Supplementary Figure 14. Enzymatic kinetics of CuN_x-SAzymes and CuO_x nanozymes for TMB substrate at pH 3.54. These data are presented as mean values ± SD (n = 3 independent experiments).

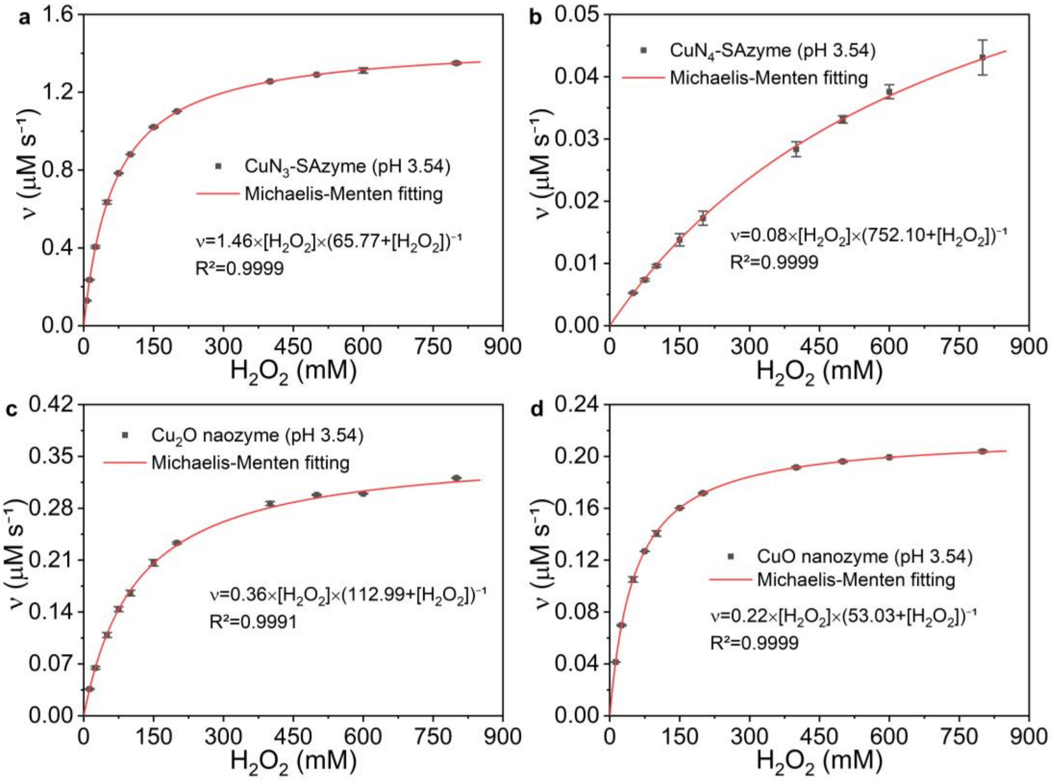

Supplementary Figure 15. Enzymatic kinetics of CuN_x-SAzymes and CuO_x nanozymes for H_2O_2 substrate at pH 3.54. These data are presented as mean values ± SD (n = 3 independent experiments).

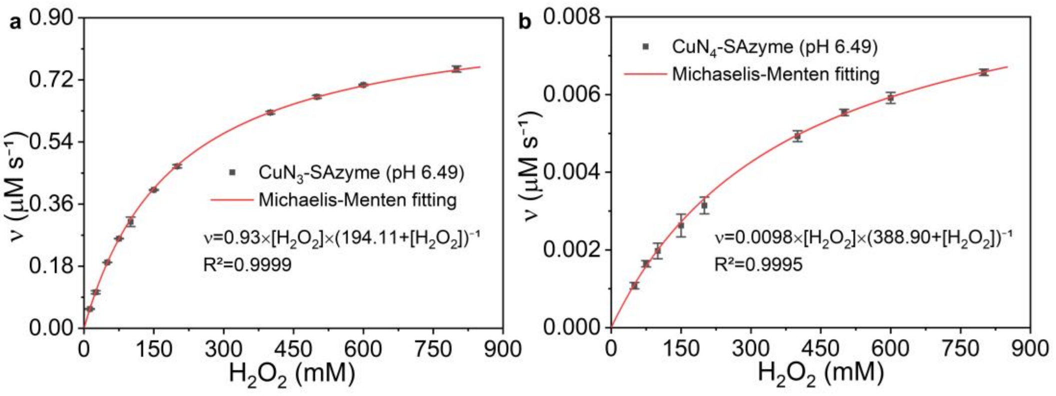

Supplementary Figure 16. Enzymatic kinetics of CuN_x-SAzymes for H_2O_2 substrate at pH 6.49. These data are presented as mean values ± SD (n = 3 independent experiments).

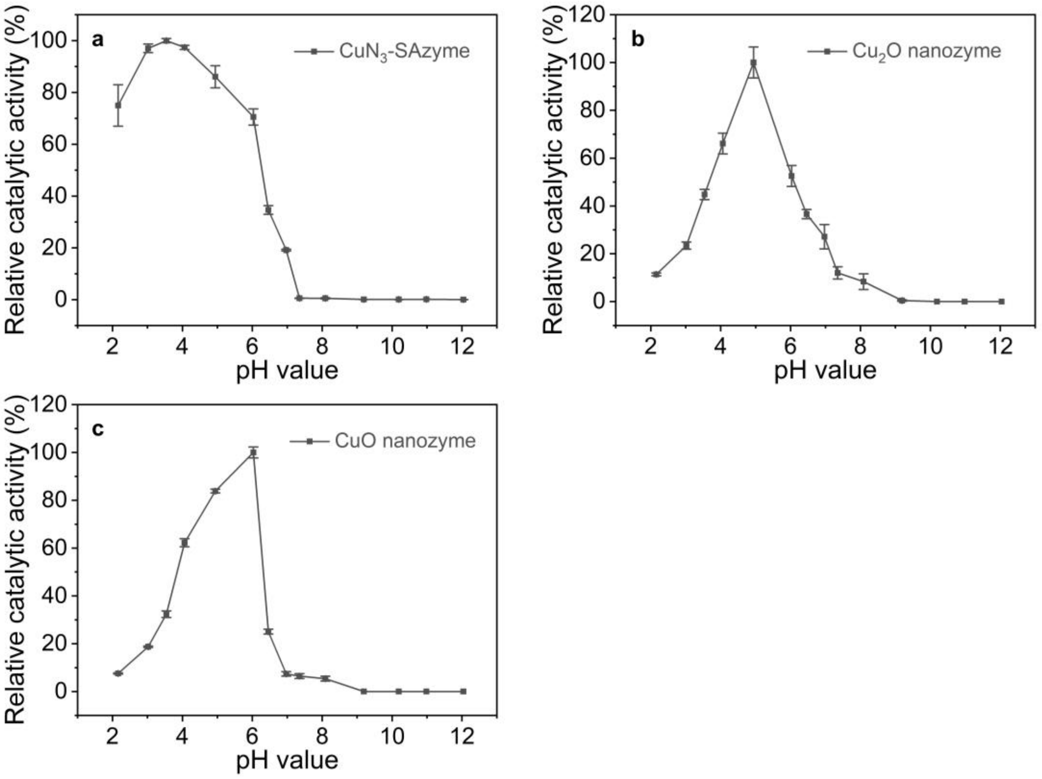

Supplementary Figure 17. Effects of pH on the enzymatic activity of CuN_3-SAzyme and CuO_x nanozymes. These data are presented as mean values ± SD (n = 3 independent experiments).

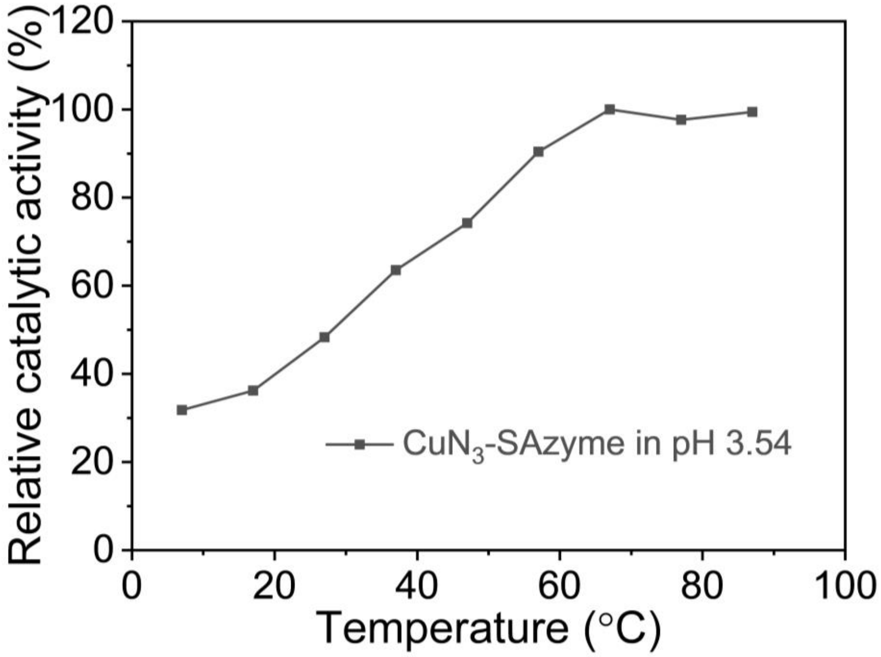

Supplementary Figure 18. Effect of temperature on the enzymatic activity of CuN_3-SAzyme. These data are presented as mean values ± SD (n = 3 independent experiments).

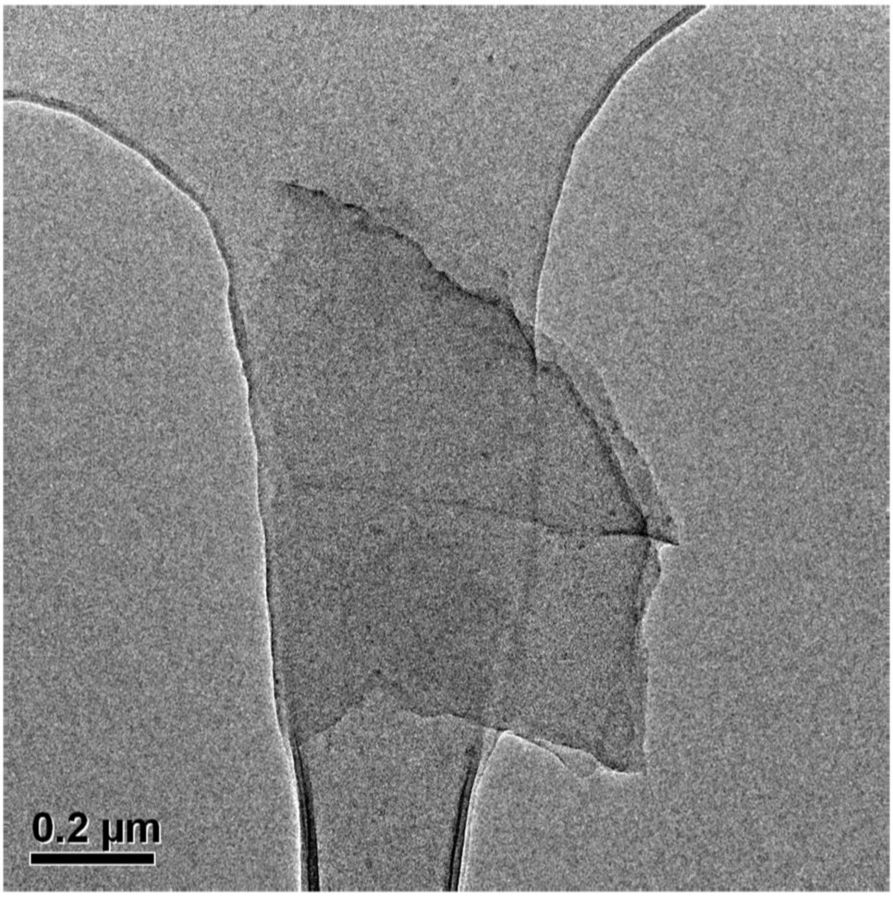

Supplementary Figure 19. TEM image of CuN_3-SAzyme treated at 60 ^{o}C for 24 h (pH 3.54). Three times each morphology characterization was repeated independently with similar results. Representative images are presented.

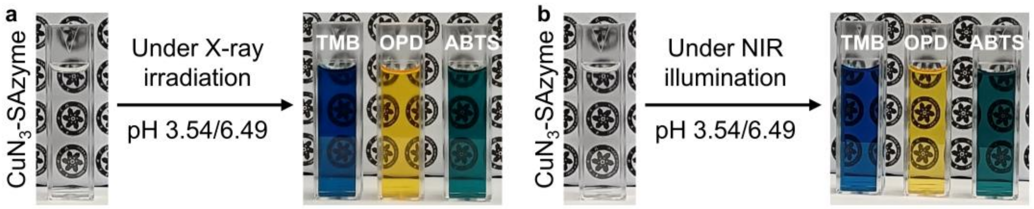

Supplementary Figure 20. Photographs of peroxidase substrate (TMB, OPD, and ABTS) solutions catalyzed by CuN_3-SAzyme under X-ray (a) and 808 nm NIR light (b) irradiation.

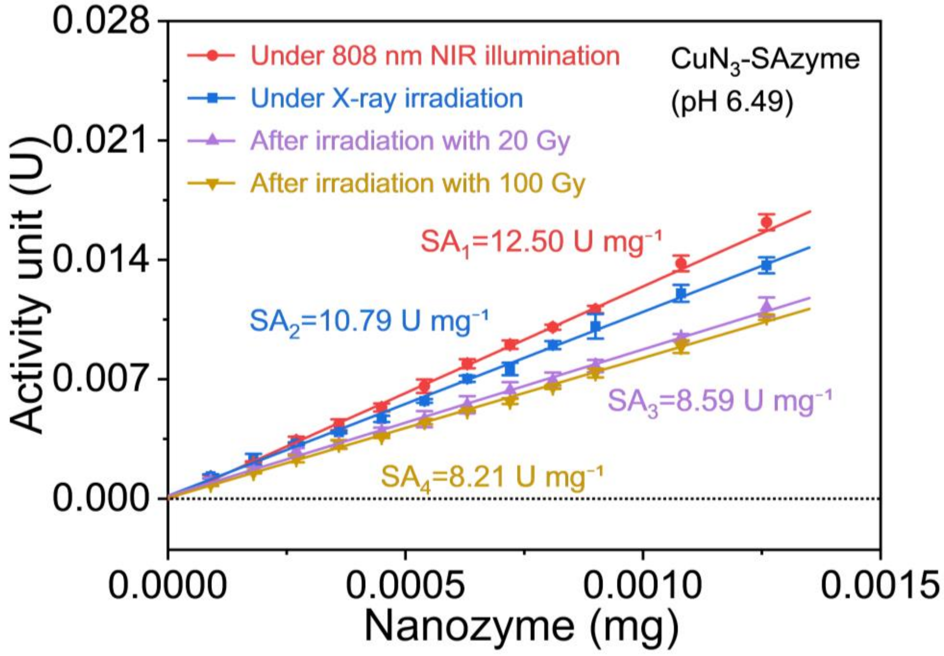

Supplementary Figure 21. Specific activities of CuN_3-SAzyme under 808 nm NIR light/X-ray irradiation or after irradiation by X-ray with the radiation dose of 20 Gy/100 Gy (pH 6.49). These data are presented as mean values ± SD (n = 3 independent experiments).

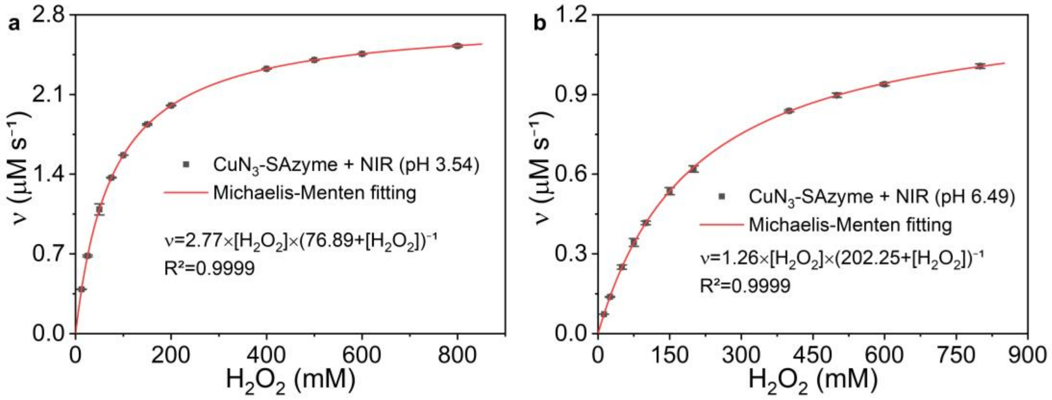

Supplementary Figure 22. Enzymatic kinetics of CuN_3-SAzyme for H_2O_2 substrate at pH 3.54 (**a**) and pH 6.49 (**b**) under 808 nm NIR light illumination. These data are presented as mean values ± SD (n = 3 independent experiments).

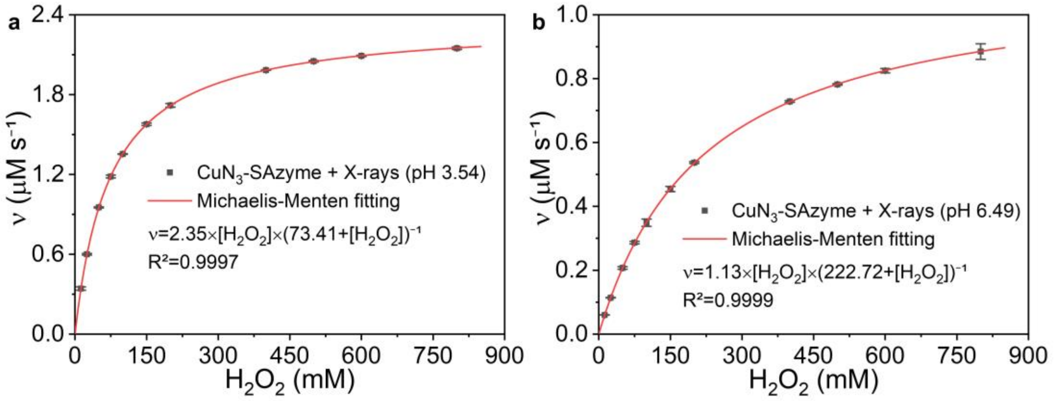

Supplementary Figure 23. Enzymatic kinetics of CuN_3-SAzyme for H_2O_2 substrate at pH 3.54 (**a**) and pH 6.49 (**b**) under X-ray irradiation. These data are presented as mean values ± SD (n = 3 independent experiments).

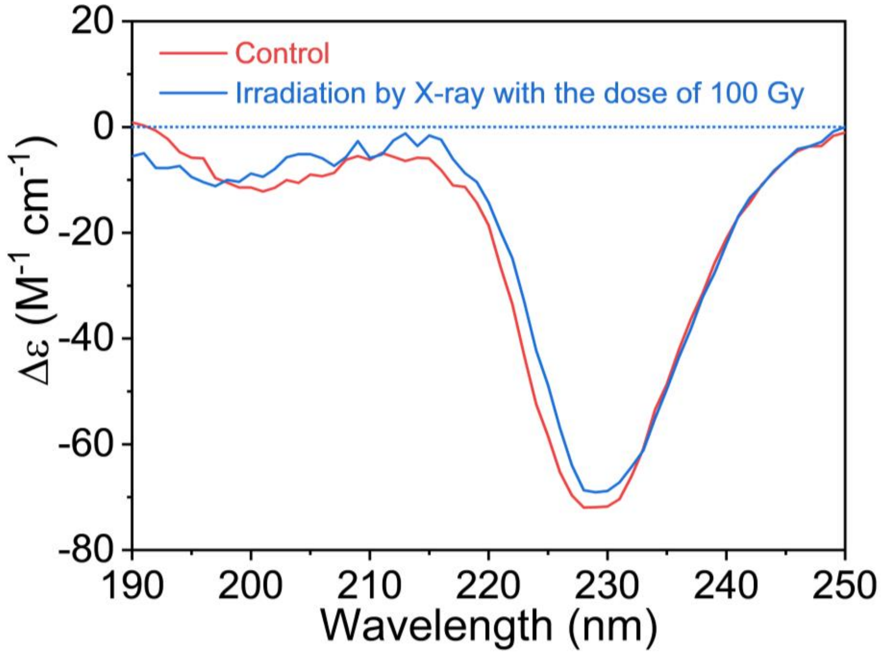

Supplementary Figure 24. Circular dichroism spectra of natural HRP irradiated by X-ray with the radiation dose of 100 Gy.

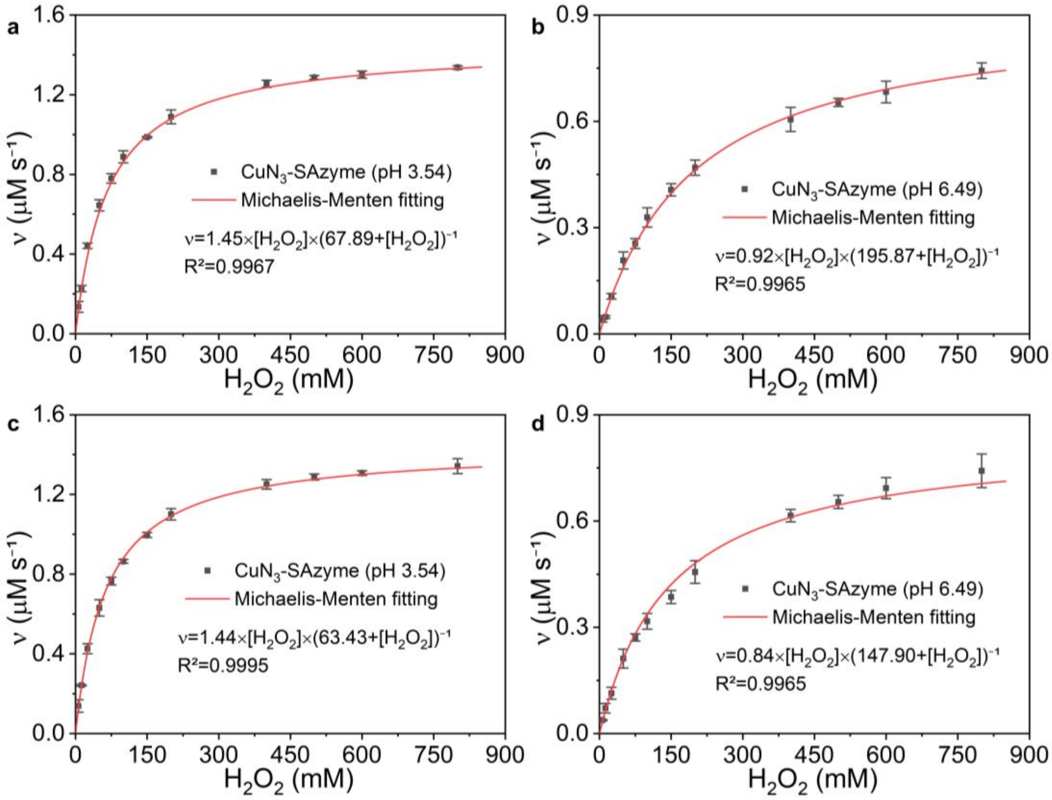

Supplementary Figure 25. **a,b** Enzymatic kinetics of CuN_3-SAzyme irradiated by X-ray with the radiation dose of 20 Gy for H_2O_2 substrate at pH 3.54 (**a**) and pH 6.49 (**b**). **c-d** Enzymatic kinetics of CuN_3-SAzyme irradiated by X-ray with the radiation dose of 100 Gy for H_2O_2 substrate at pH 3.54 (**c**) and pH 6.49 (**d**). These data are presented as mean values ± SD (n = 3 independent experiments).

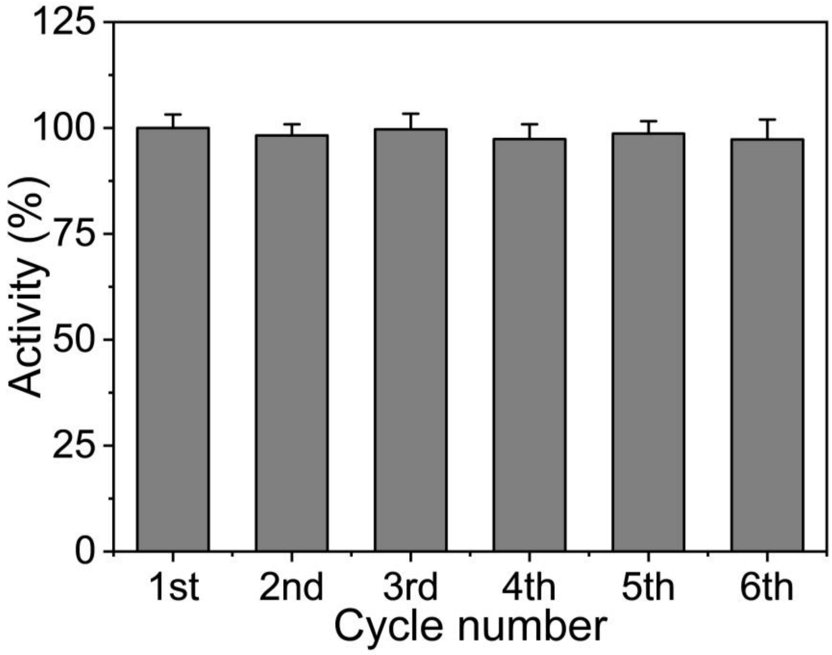

Supplementary Figure 26. Evaluation of the enzymatic activity of CuN_3-SAzyme under X-ray irradiation, after up to six cycles. These data are presented as mean values ± SD (n = 3 independent experiments).

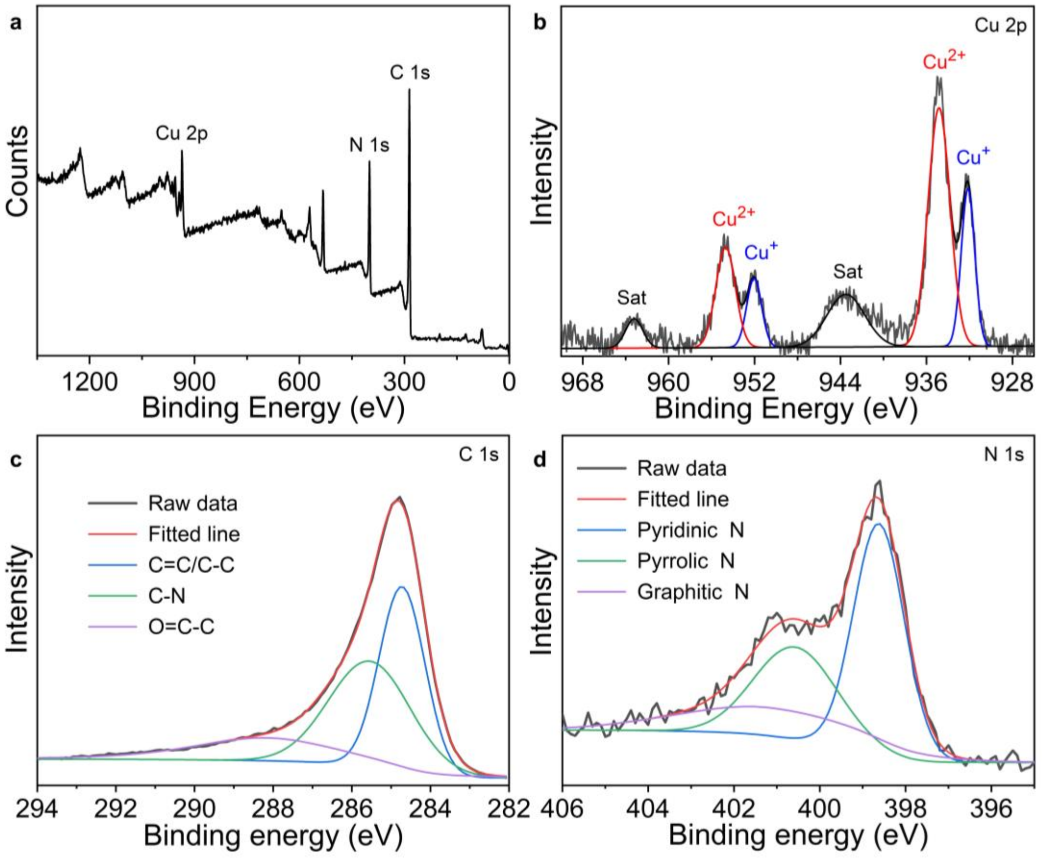

Supplementary Figure 27. XPS analysis of CuN_3-SAzyme after X-ray irradiation. a XPS survey spectrum. **b-d** Cu 2p (**b**), C 1s (**c**), and N 1s (**d**) XPS spectra.

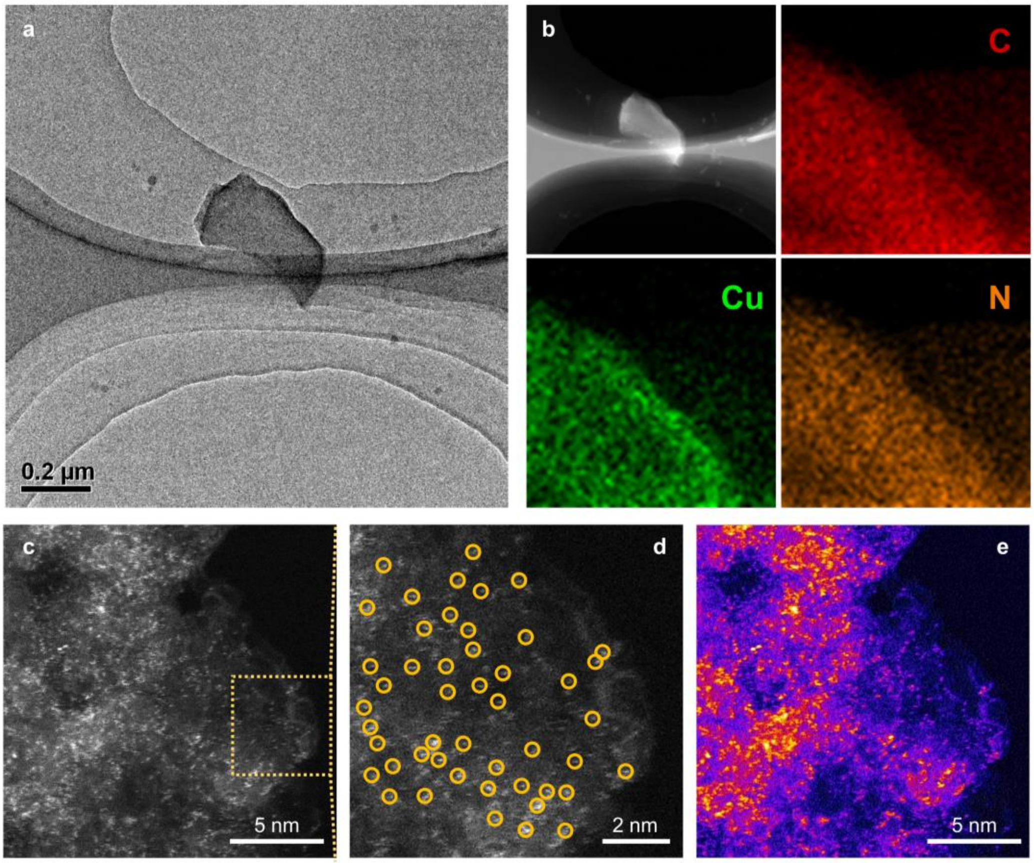

Supplementary Figure 28. Characterization of CuN_3-SAzyme after irradiation by X-ray with the radiation dose of 100 Gy. a HR-TEM image. **b** HAADF-STEM image and corresponding EDS mapping. **c** Atomic-level HAADF-STEM image. **d** Enlarged HAADF-STEM image of the marked area in ***c***. **e** Corresponding surface intensity map of ***c***, the yellow dots are Cu atoms. Three times each morphology characterization was repeated independently with similar results. Representative images are presented.

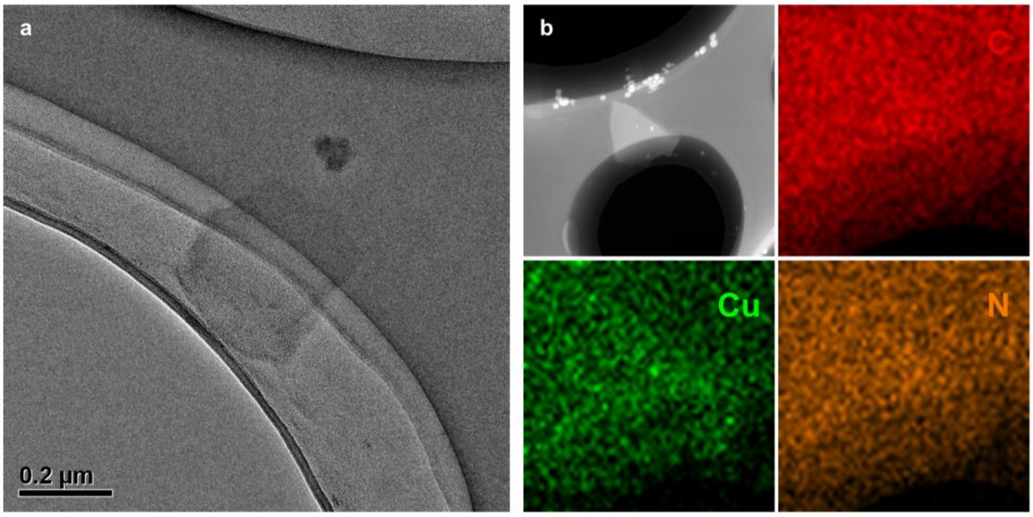

Supplementary Figure 29. Characterization of CuN_3-SAzyme after irradiation by X-ray with the radiation dose of 500 Gy. a HR-TEM image. **b** HAADF-STEM image and corresponding EDS mapping. Three times each morphology characterization was repeated independently with similar results. Representative images are presented.

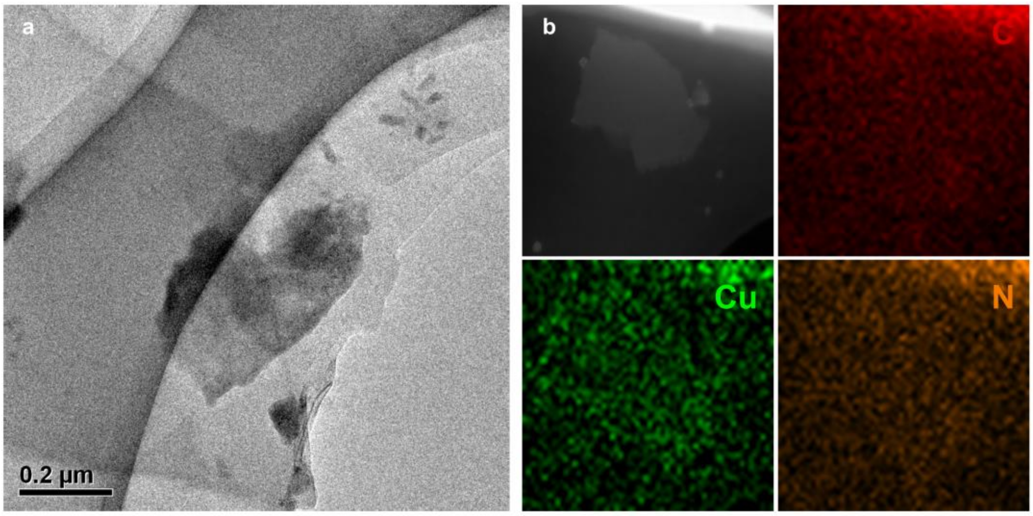

Supplementary Figure 30. Characterization of CuN_3-SAzyme after repeated irradiation by X-ray. a TEM image. **b** HAADF-STEM image and corresponding EDS mapping. Three times each morphology characterization was repeated independently with similar results. Representative images are presented.

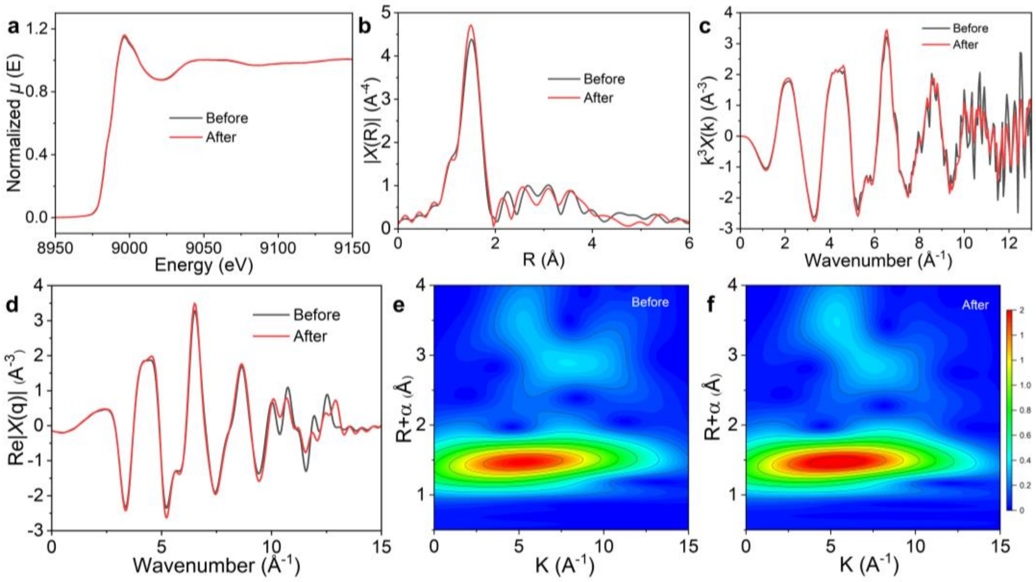

Supplementary Figure 31. Comparison of XAFS spectra of CuN_3-SAzyme before/after X-ray irradiation (200 Gy). a Cu K-edge XANES spectra. **b** Fourier-transformed magnitudes of experimental Cu K-edge EXAFS signals at *R* space. **c** *k*-space plots. **d** *q*-space plots. **e,f** WT curves.

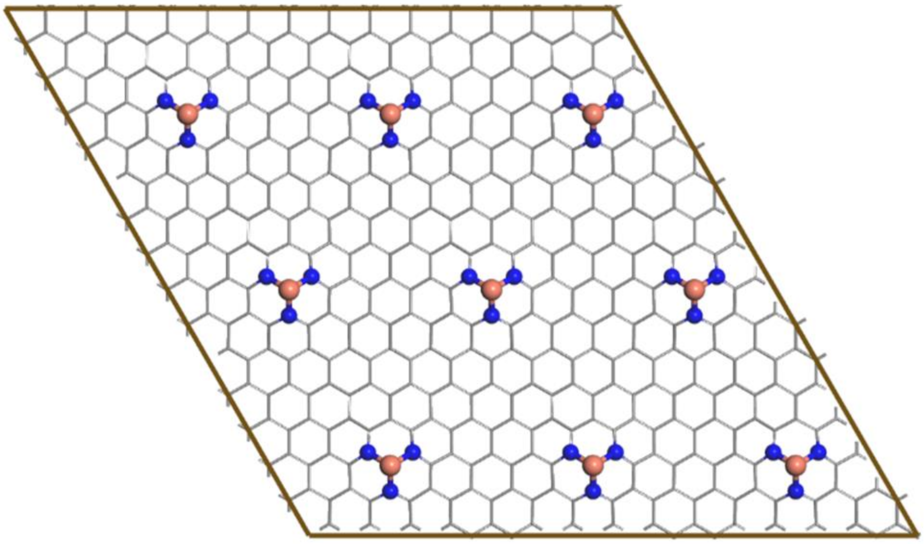

Supplementary Figure 32. Simulation model used for the AIMD simulations, containing a total of 414 carbon, 27 nitrogen, and 9 copper atoms. Gray: C; Blue: N; Light coral: Cu. The boundary of the supercell is shown in pinkish brown.

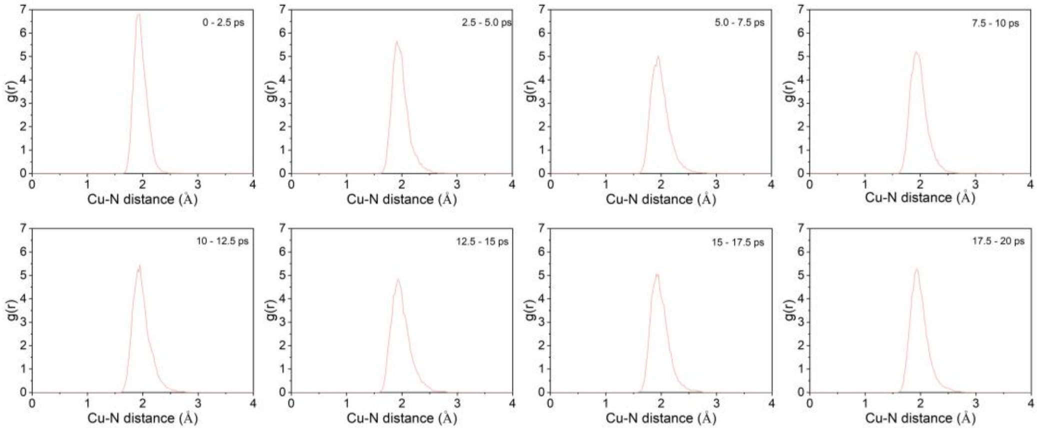

Supplementary Figure 33. Time evolution of the distribution function g(r) for the distance between Cu and N atoms in different time periods during the AIMD simulations at 1,773 K. It is obvious that the Cu-N bonds can be well maintained.

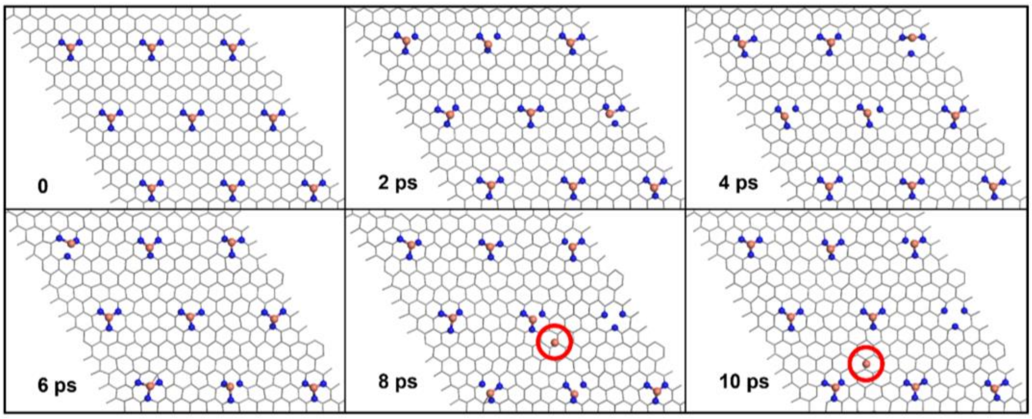

Supplementary Figure 34. Snapshots of the CuN_3-SAzyme model at different moments in the AIMD simulations at 2,273 K. One of the Cu atoms has detached from the N3 group in the second half of the simulation (red circles). The disruption of CuN_3 active moiety becomes more pronounced as the temperature is further increased to 2,773 K and 3,273 K.

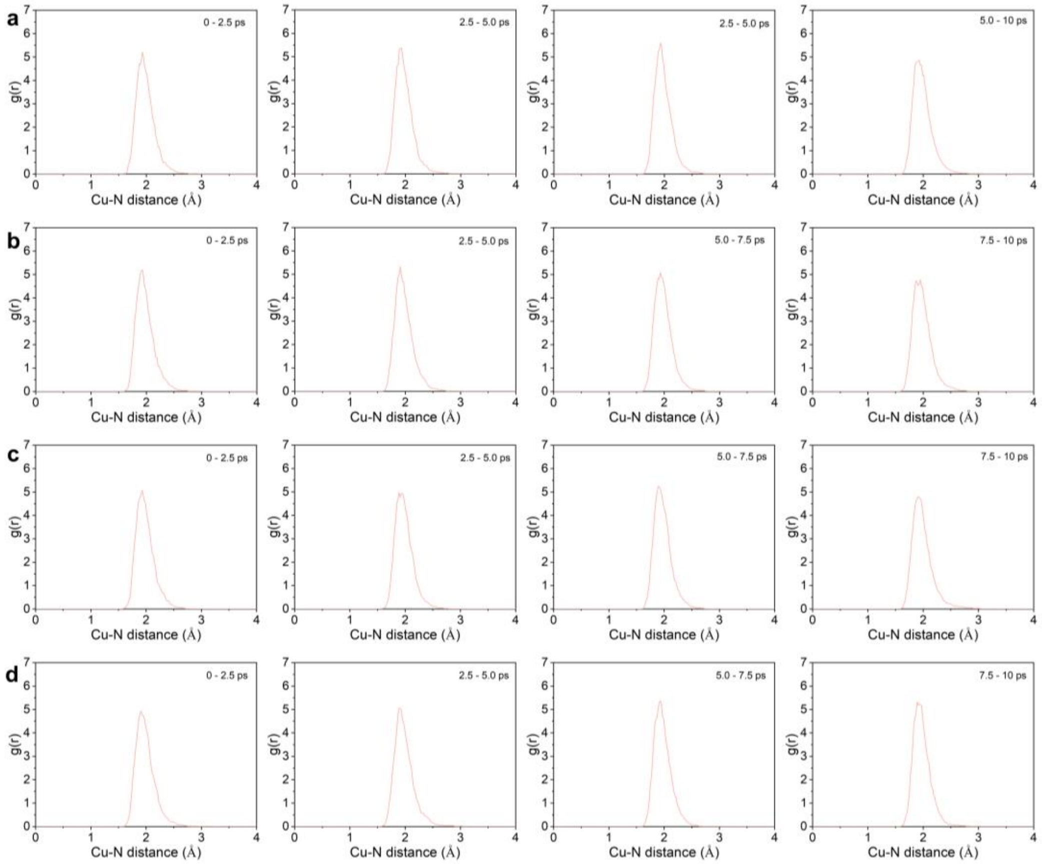

Supplementary Figure 35. Time evolution of the distribution function *g(r)* for the distance between Cu and N atoms in different time periods during the AIMD simulations at 1,773 K after one (**a**), two (**b**), three (**c**), and four (**d**) electrons have been ionized. The ionization occurs after the AIMD simulations at 1,773 K have run for 5 ps in Supplementary Fig. S34. It is obvious that Cu-N bonds are still well maintained.

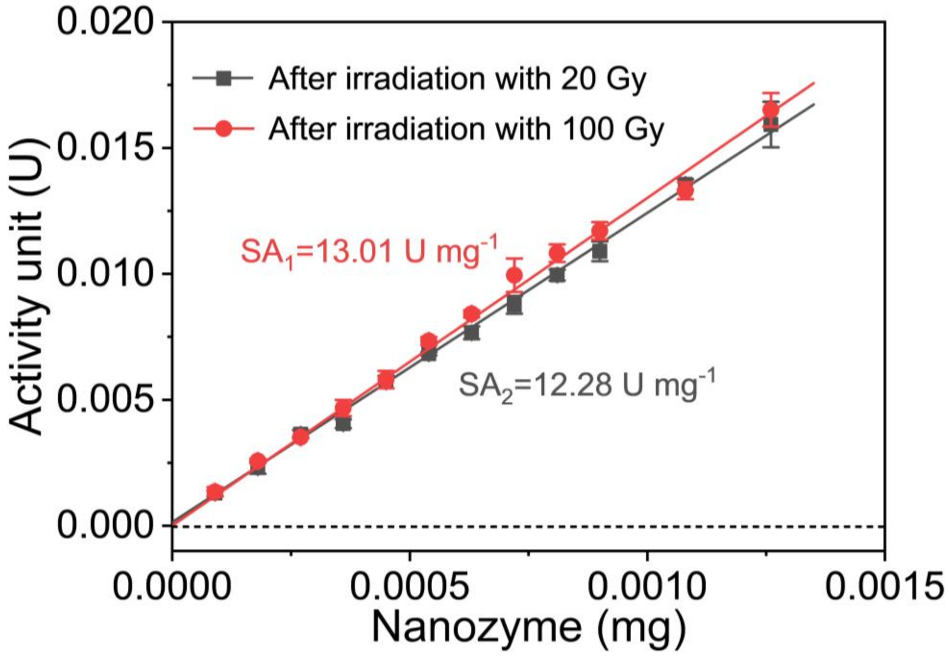

Supplementary Figure 36. Evaluation of the specific activities of CuN_3-SAzyme after irradiation by γ-ray with the radiation dose of 20 Gy/100 Gy (pH 3.54). These data are presented as mean values ± SD (n = 3 independent experiments).

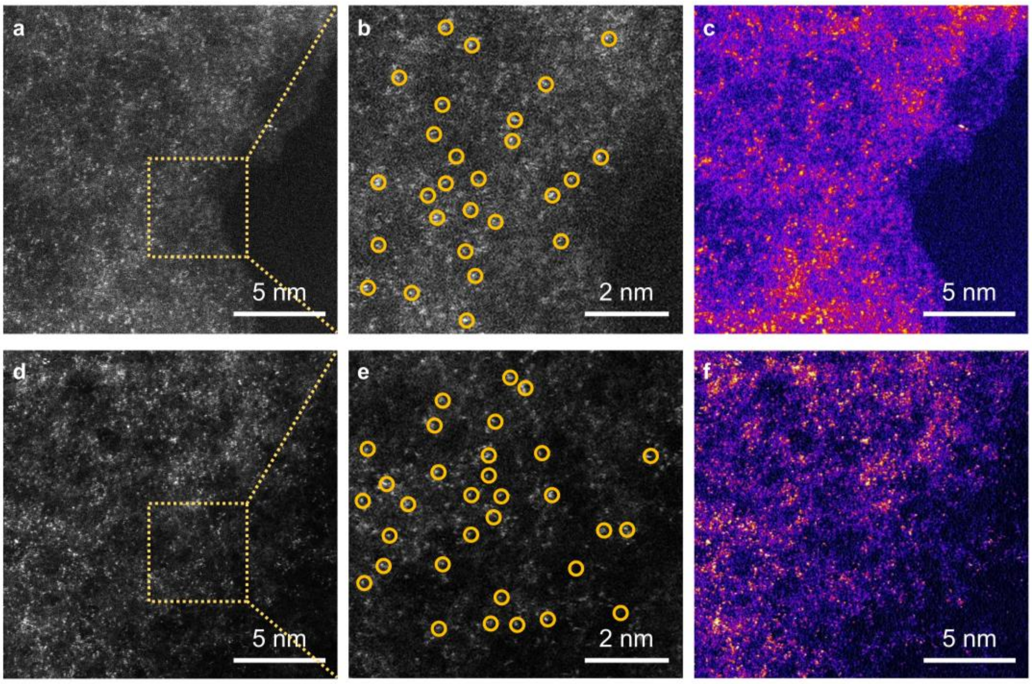

Supplementary Figure 37. Characterization of CuN_3-SAzyme after irradiation by γ-ray with the radiation dose of 100 Gy (**a-c**) and 500 Gy(**d-f**). **a,d** Atomic-level HAADF-STEM image. **b,e** Enlarged HAADF-STEM image of the marked area in ***a*** (**b**) or ***d*** (**e**). **c,f** Corresponding surface intensity map of ***a*** (c) or ***d*** (f). The yellow dots are Cu atoms. Three times each morphology characterization was repeated independently with similar results. Representative images are presented.

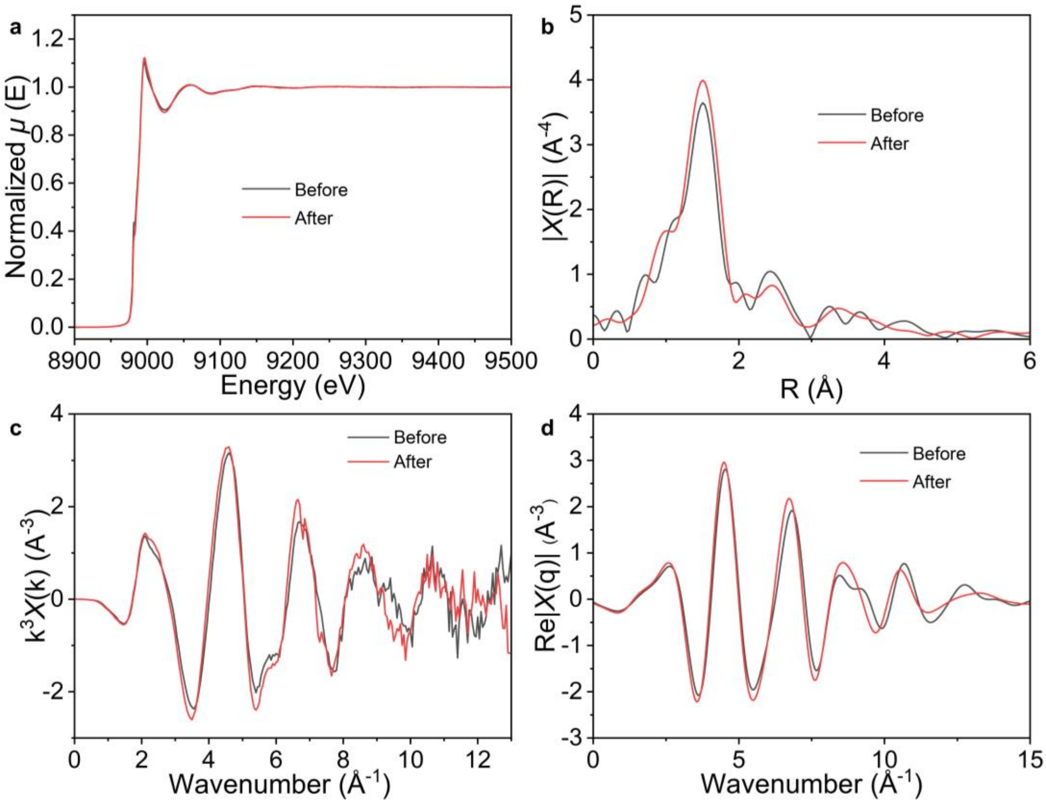

Supplementary Figure 38. Comparison of XAFS spectra of CuN_3-SAzyme before/after γ-ray irradiation (500 Gy). **a** Cu K-edge XANES spectra. **b** Fourier-transformed magnitudes of experimental Cu K-edge EXAFS signals at *R* space. **c** *k*-space plots. **d** *q*-space plots.

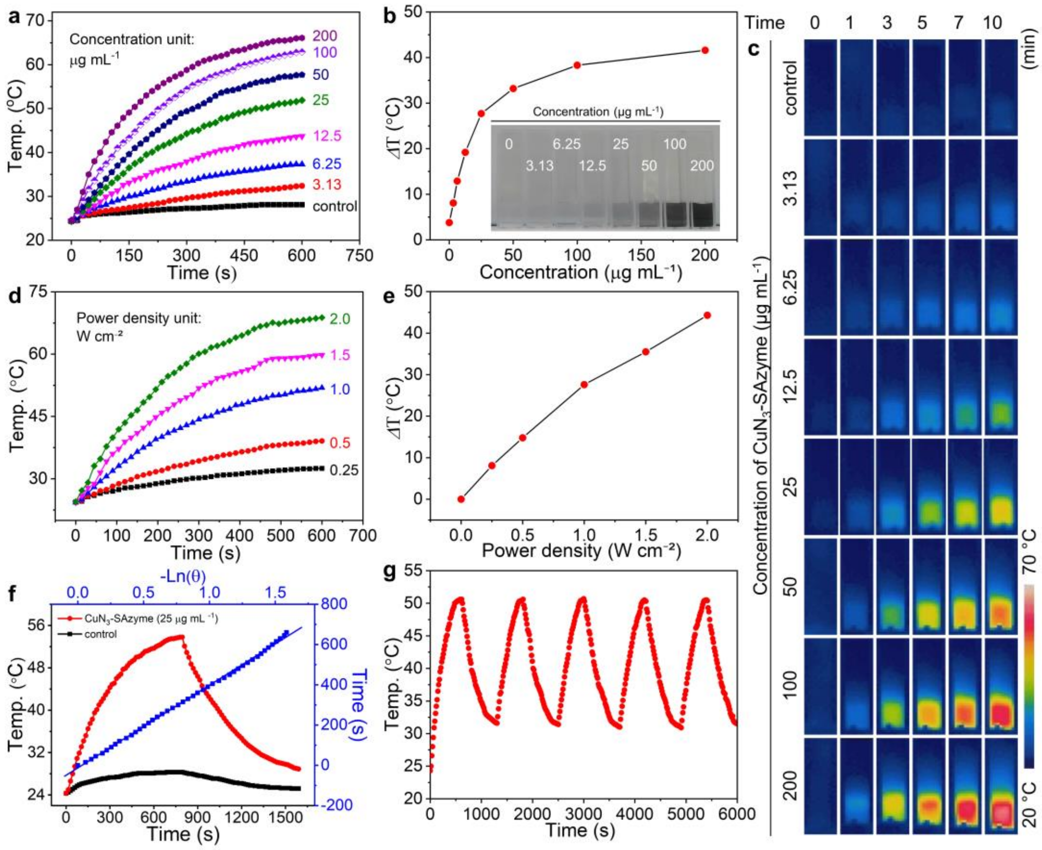

Supplementary Figure 39. a Temperature elevation curves of aqueous suspensions of CuN_3-SAzyme under 808 nm NIR light illumination. **b** Plot of temperature change versus the concentration of CuN_3-SAzyme. Inset: Photograph of aqueous suspensions of CuN_3-SAzyme. **c** Corresponding infrared thermal images of aqueous suspensions of CuN_3-SAzyme. **d** Temperature elevation curves of aqueous suspensions of CuN_3-SAzyme illuminated by 808 nm NIR light with various power densities. **e** Plot of temperature change versus the power density of 808 nm NIR light. **f** Left axis: Heating and cooling curves; Right axis: Linear time data versus -Ln(θ). **g** Photothermal profiles of aqueous suspensions of CuN_3-SAzyme over five cycles.

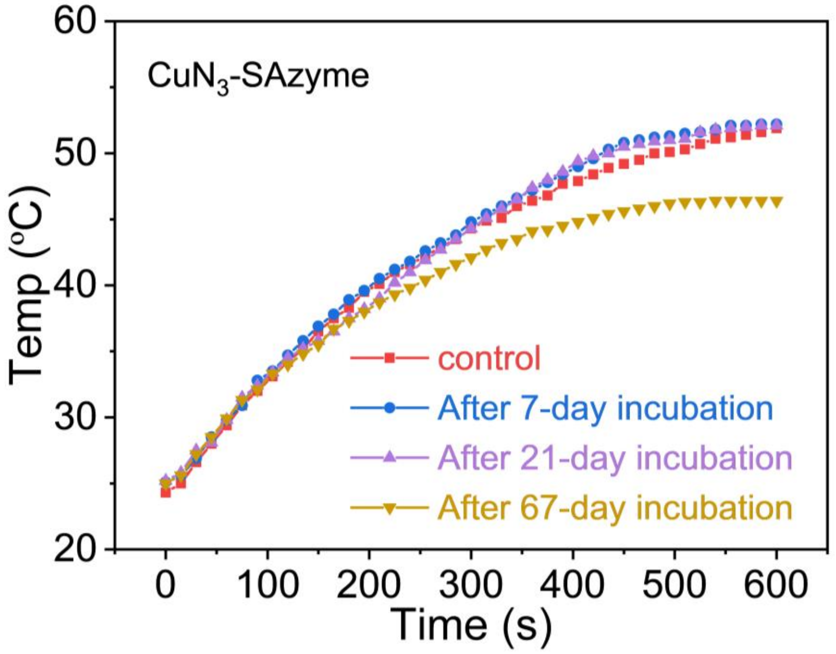

Supplementary Figure 40. Temperature elevation curves of aqueous suspensions of CuN_3-SAzyme after incubation in DMEM for various days.

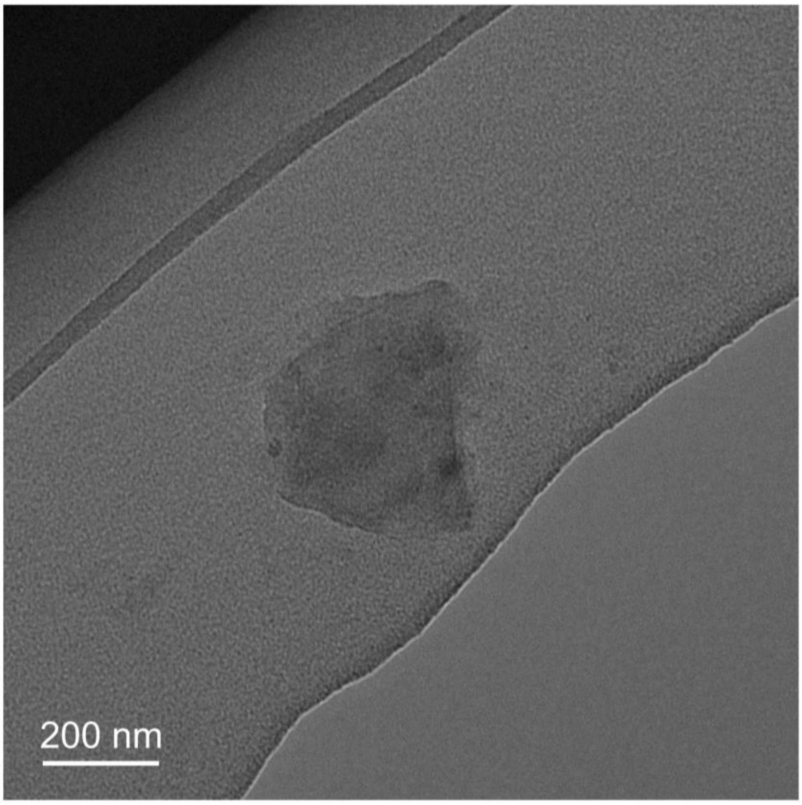

Supplementary Figure 41. TEM image of nanoscale CuN_3-SAzyme. Three times each morphology characterization was repeated independently with similar results. Representative images are presented.

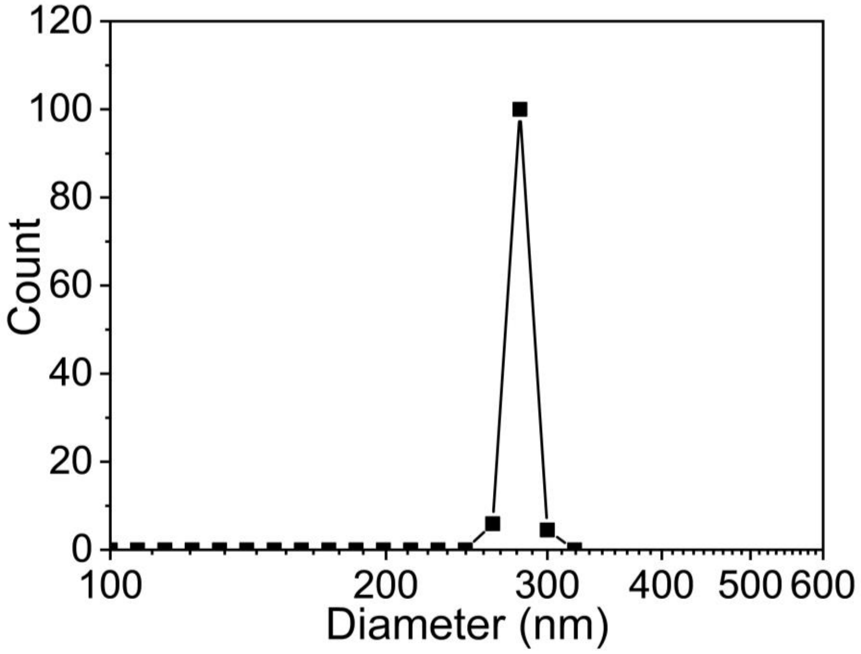

Supplementary Figure 42. Average hydrodynamic diameter of nanoscale CuN_3-SAzyme.

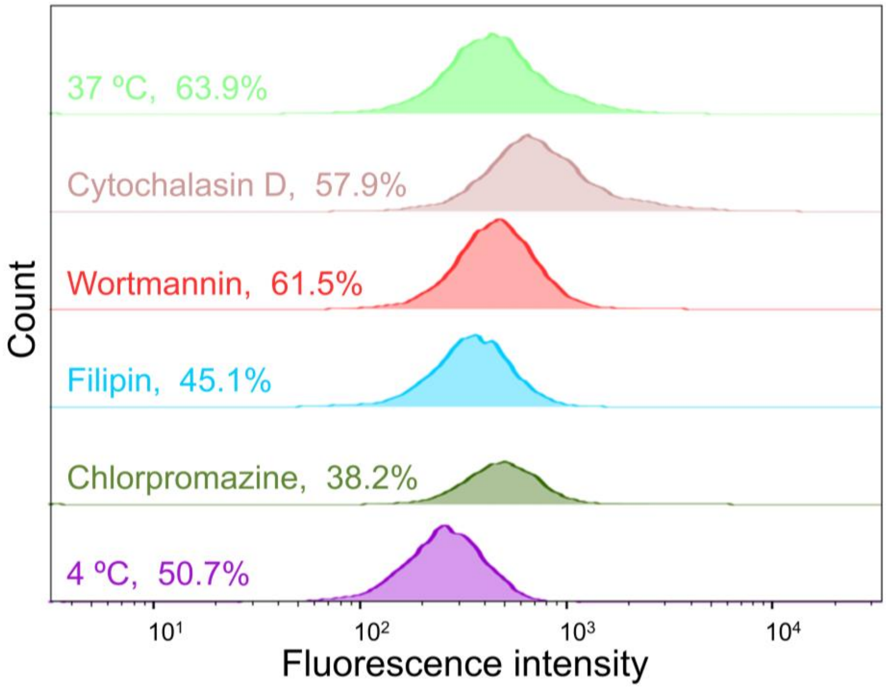

Supplementary Figure 43. Flow cytometry histograms illustrating cellular uptake and inhibition of uptake of CuN_3-SAzyme.

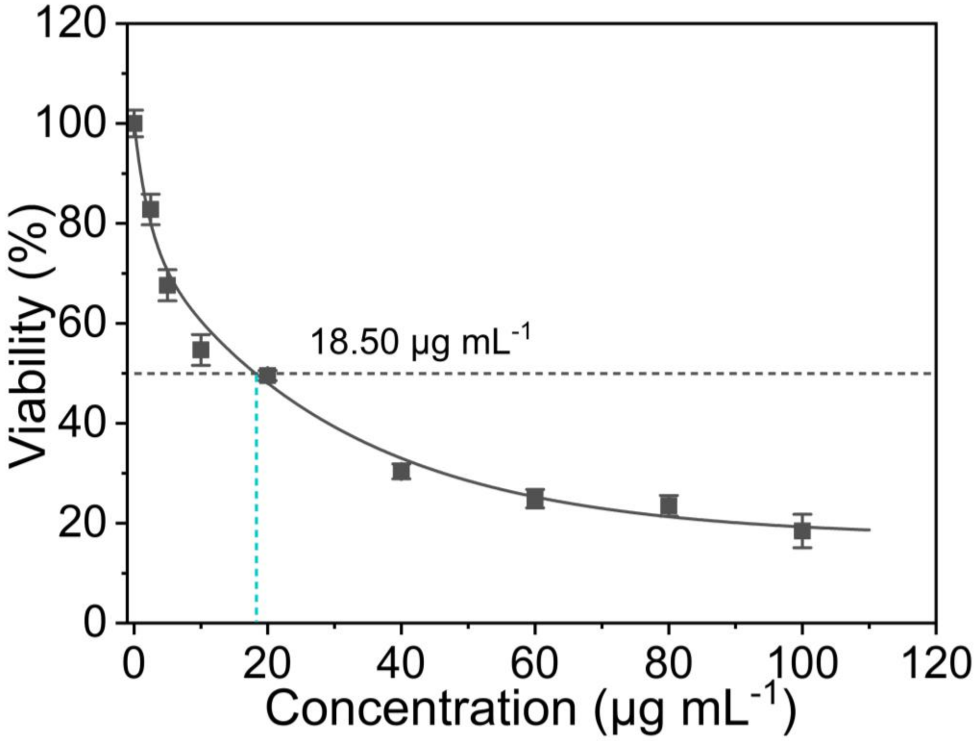

Supplementary Figure 44. Viability of K7M2 cells after incubation with CuN_3-SAzyme. These data are presented as mean values ± SD (n = 6 independent experiments).

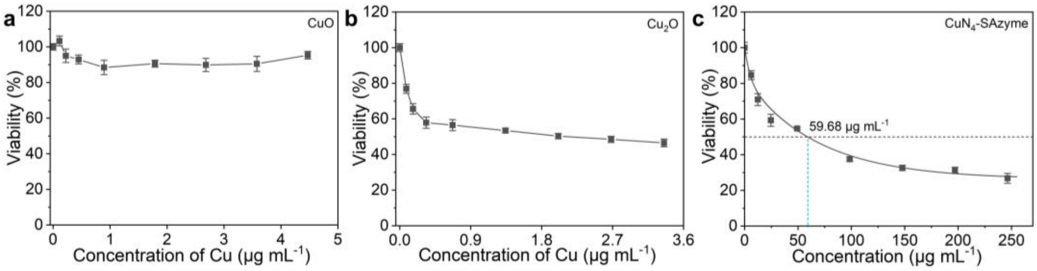

Supplementary Figure 45. Viabilities of 4T1 cells after incubation with CuO nanozyme (**a**), Cu_2O nanozyme (**b**), and CuN_4-SAzyme (**c**). These data are presented as mean values ± SD (n = 6 independent experiments).

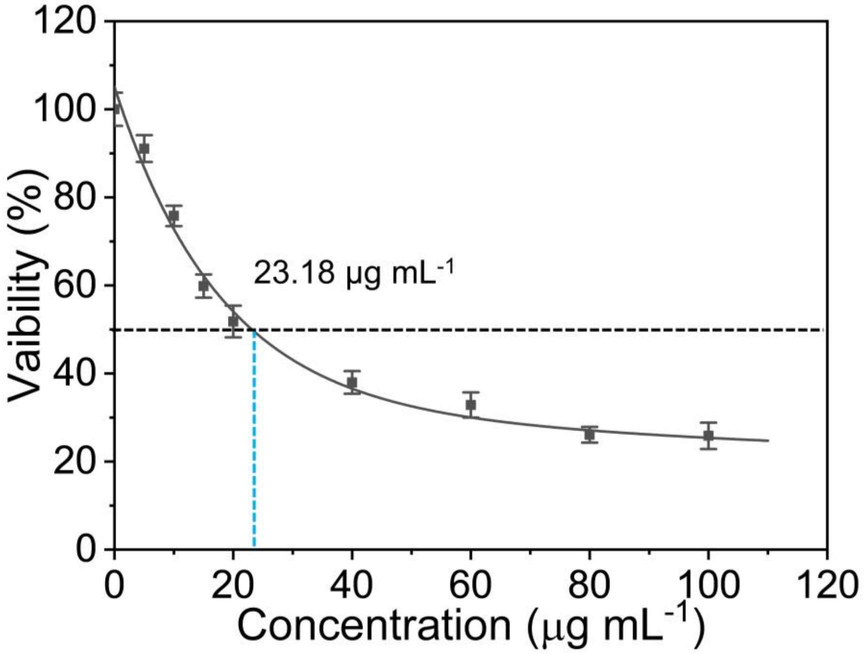

Supplementary Figure 46. Viability of 3T3 cells after incubation with CuN_3-SAzyme. These data are presented as mean values ± SD (n = 6 independent experiments).

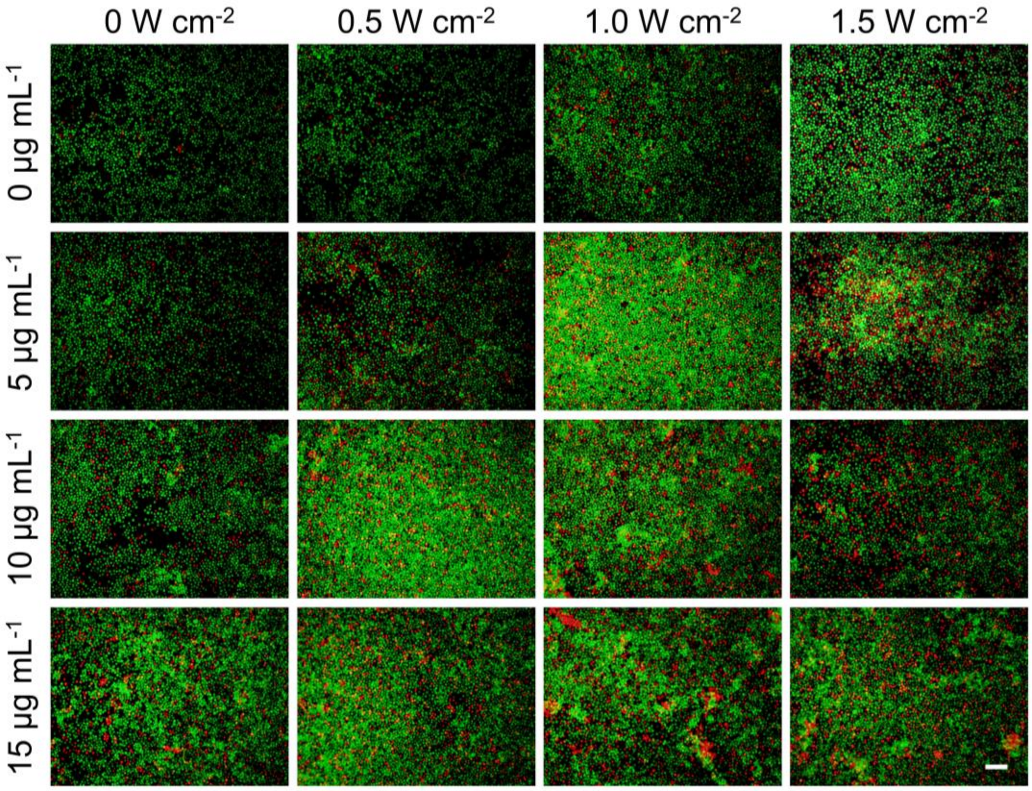

Supplementary Figure 47. Live/Dead staining of 4T1 cells incubated with CuN_3-SAzyme and then illuminated with 808 nm NIR light. Experiments were performed three times with similar results. Representative images are presented. Scale bar: 100 μm.

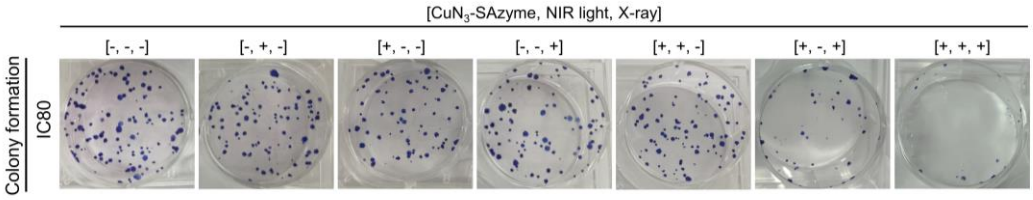

Supplementary Figure 48. Photographs of colony-forming cells. Experiments were performed three times with similar results. Representative images are presented.

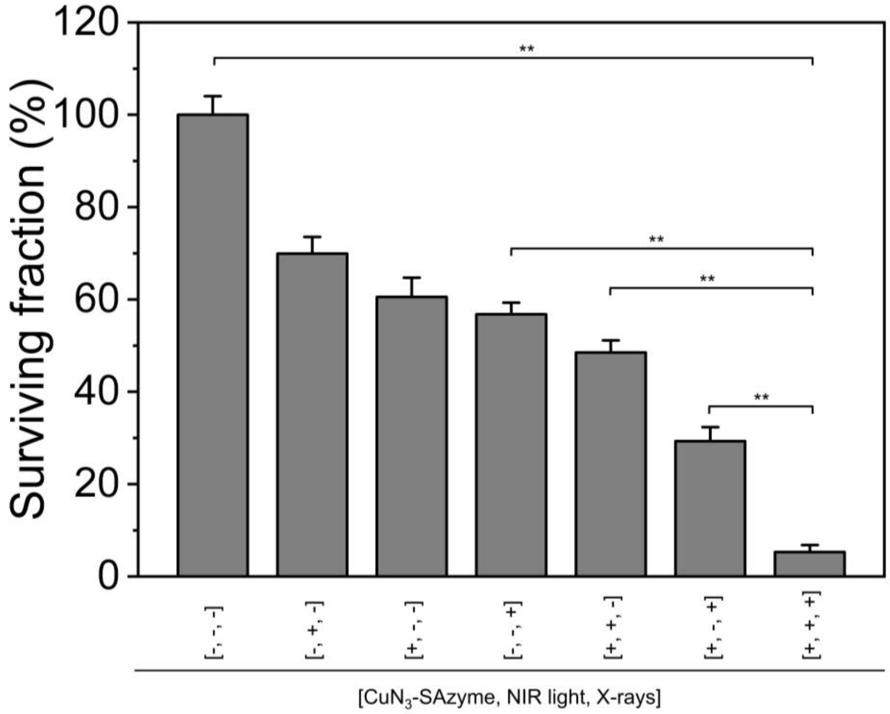

Supplementary Figure 49. Colony formation ratio of 4T1 cells with various treatments. These data are presented as mean values ± SD (n = 3 independent experiments).

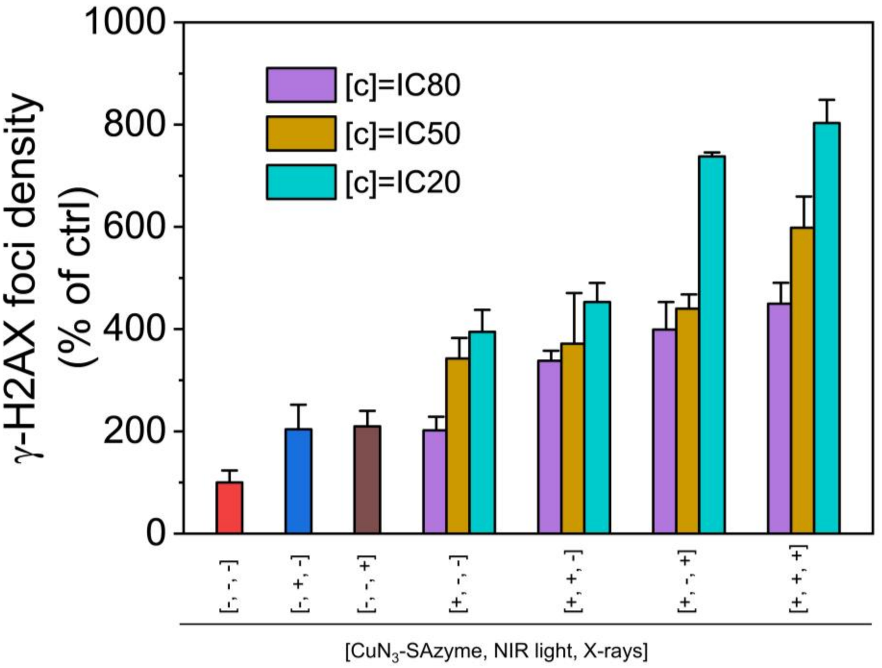

Supplementary Figure 50. Quantitative analysis of the number of γ-H2AX foci per cell. These data are presented as mean values ± SD (n = 3 independent experiments).

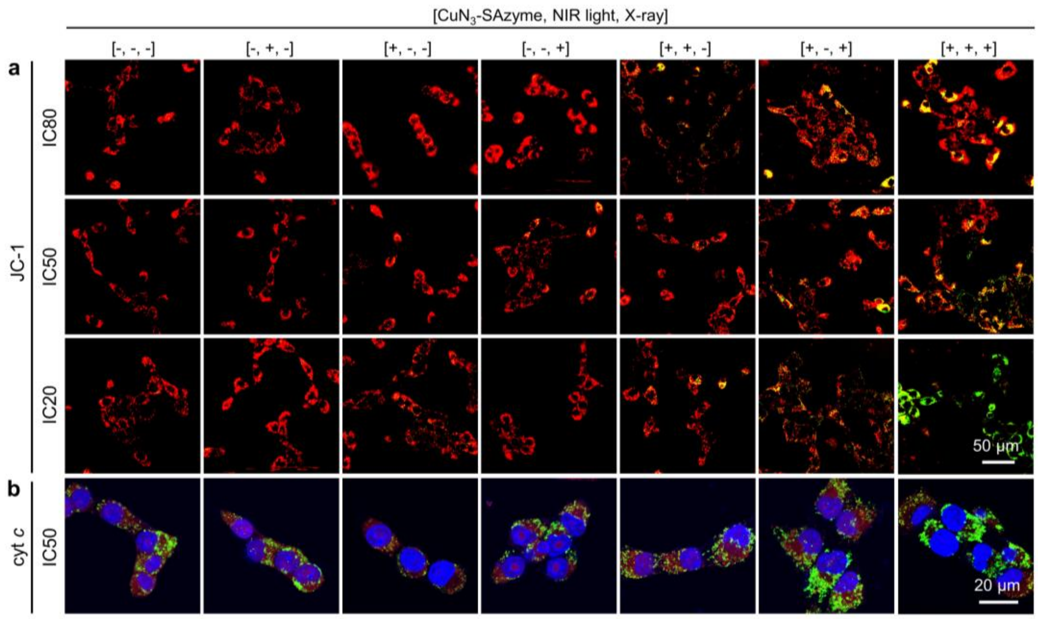

Supplementary Figure 51. a Confocal images of JC-1 stained 4T1 cells (IC80, IC50, and IC20 concentrations. **b** Colocalization analysis between mito-green and anti-cyt *c* mAbs. Experiments were performed three times with similar results. Representative images are presented.

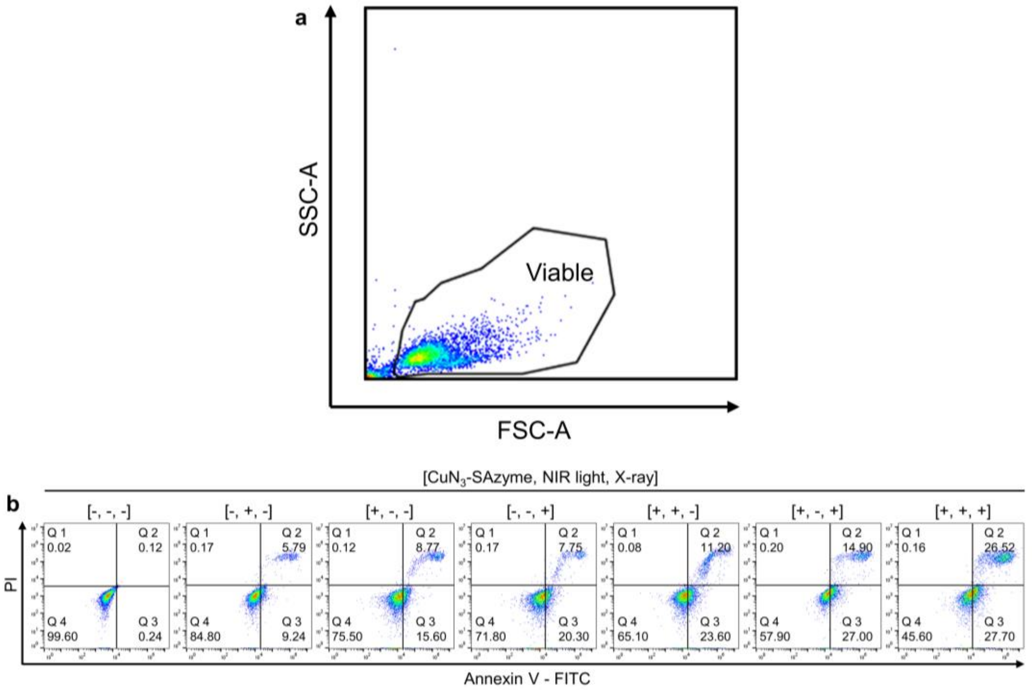

Supplementary Figure 52. a An example exemplifying the gating strategy for the flow cytometry.

b Qualitative flow cytometry data plot for 24 h.

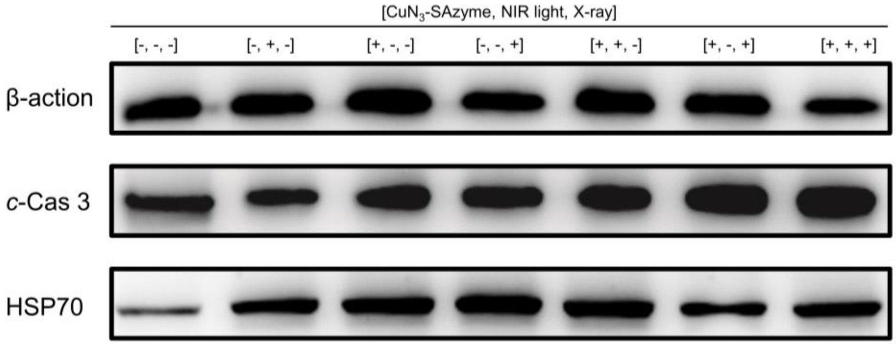

Supplementary Figure 53. Western blot analysis. Experiments were performed three times with similar results. Representative images are presented.

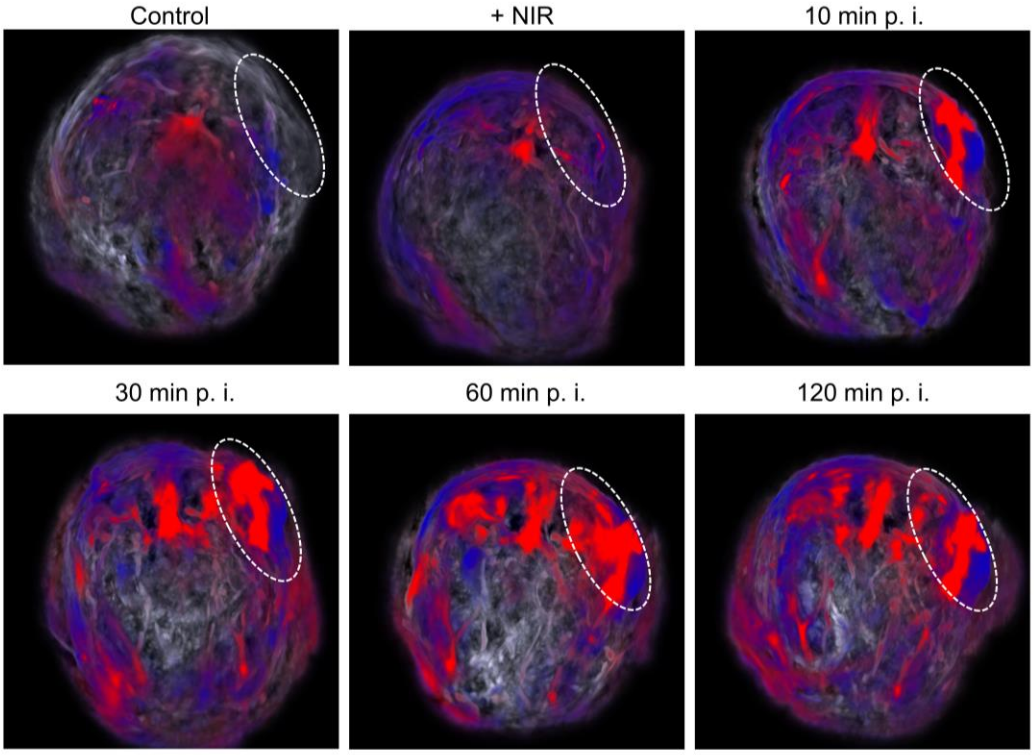

Supplementary Figure 54. In vivo three-dimension photoacoustic imaging of the tumor site after injection with CuN_3-SAzyme as a function of time. p. i.: post injection.

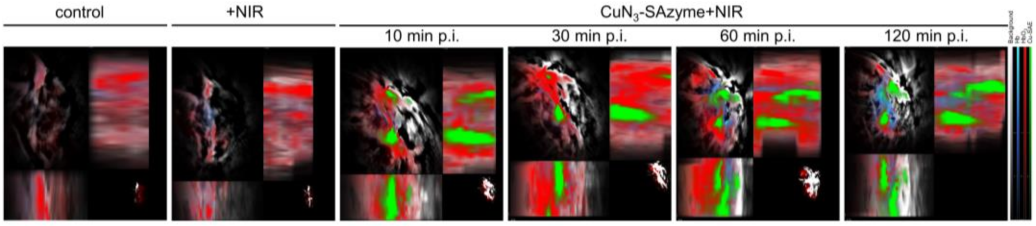

Supplementary Figure 55. In vivo three-dimension photoacoustic images of Hb (blue), HbO_2 (red), and CuN_3-SAzyme (green) after photothermal heating in the form of three views: Top left, X-Y plane maximum intensity projection (MIP) image; top right, Y-Z plane MIP image; lower left, X-Z plane MIP image; Lower right, three-dimension photoacoustic images of the reconstructed tumors.

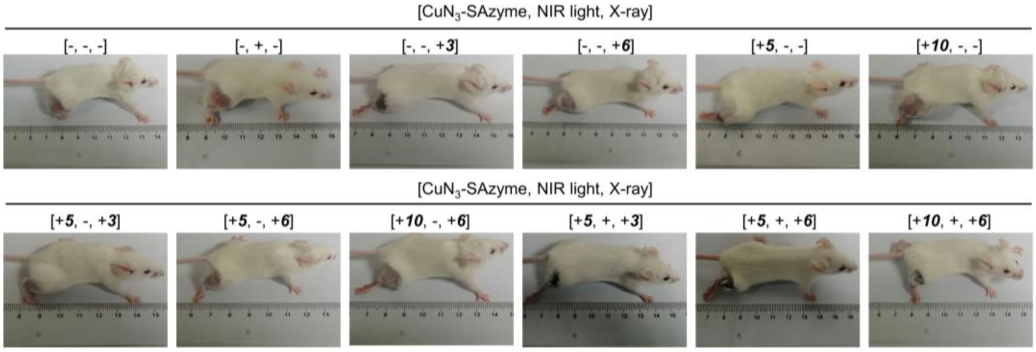

Supplementary Figure 56. Photographs of 4T1-bearing mice treated with CuN_3-SAzyme followed by 808 nm NIR light and/or X-ray irradiation at the end of treatment period.

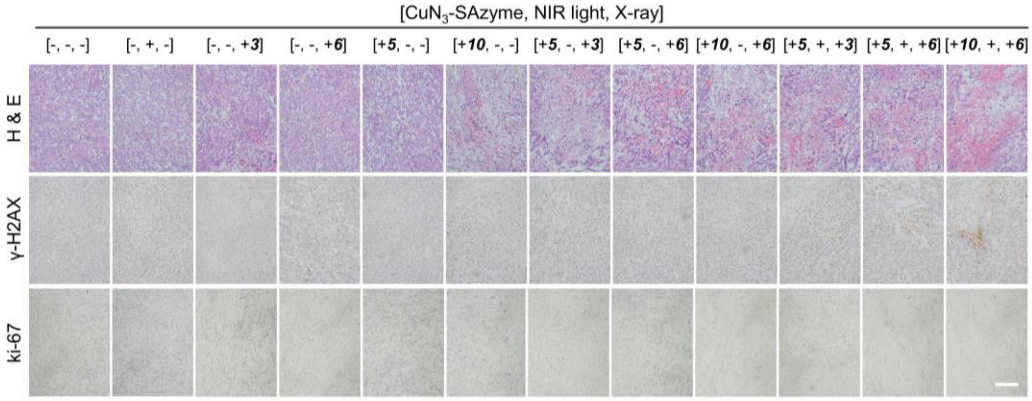

Supplementary Figure 57. Representative histopathology images of the excised tumors stained with H&E, γ-H2AX, and Ki-67 at day 3 after various treatments. Experiments were performed three times with similar results. Representative images are presented. Scale bar: 50 μm.

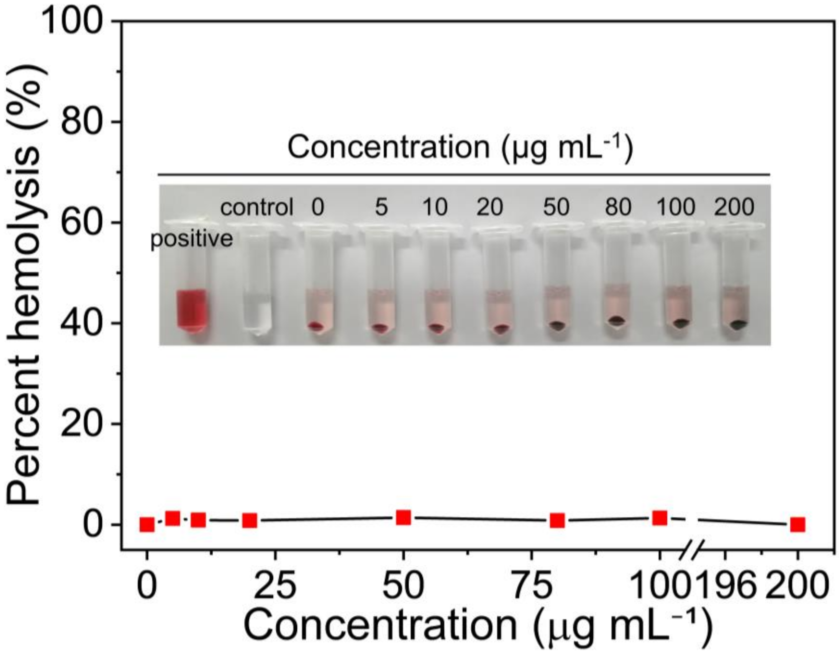

Supplementary Figure 58. In vitro hemolysis analysis of CuN_3-SAzyme. All data are presented as means ± SD (n = 3 independent experiments).

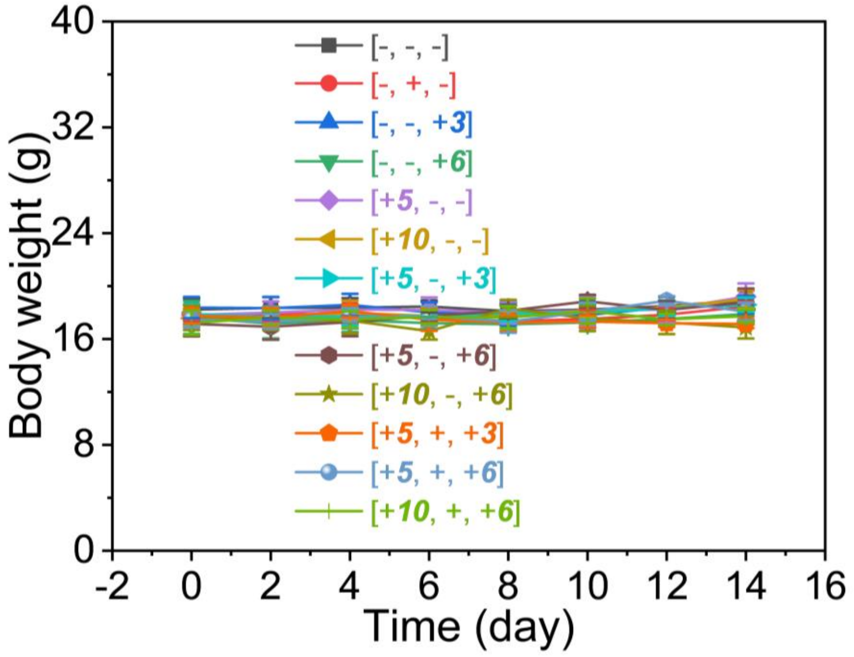

Supplementary Figure 59. Body weight of 4T1-bearing mice treated with CuN_3-SAzyme followed by 808 nm NIR light and/or X-ray irradiation. All data are presented as means ± SD (n = 5 biologically independent animals).

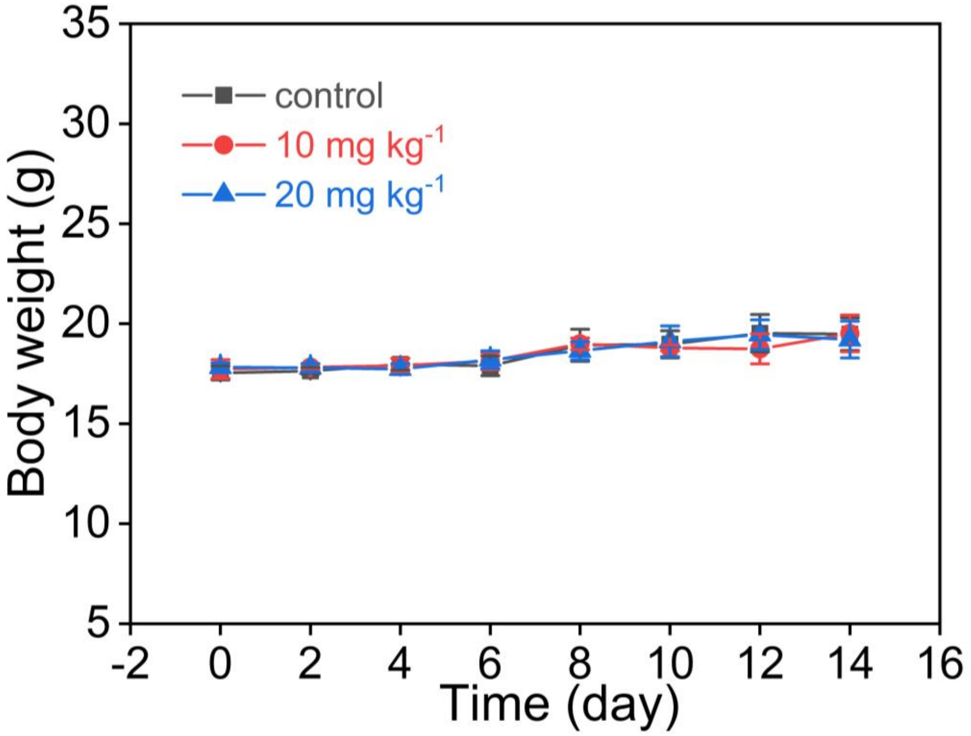

Supplementary Figure 60. Body weight of mice subcutaneously injected by CuN_3-SAzyme. All data are presented as means ± SD (n = 6 biologically independent animals).

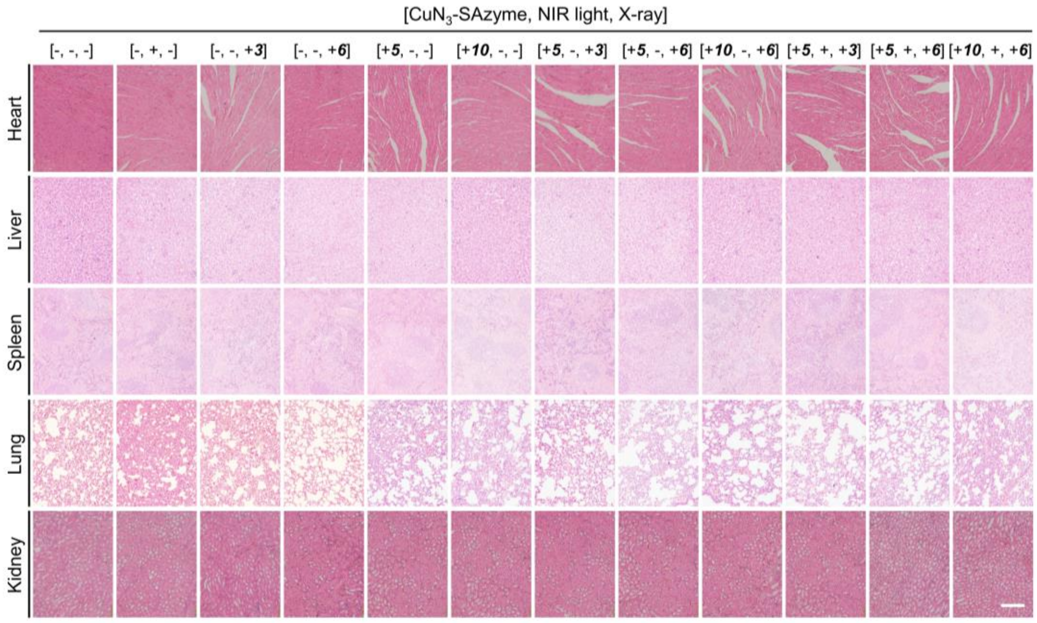

Supplementary Figure 61. Representative H&E images of main organs including heart, liver, spleen, lung, and kidney at the end of treatment period. Experiments were performed three times with similar results. Representative images are presented. Scale bar: 50 μm.

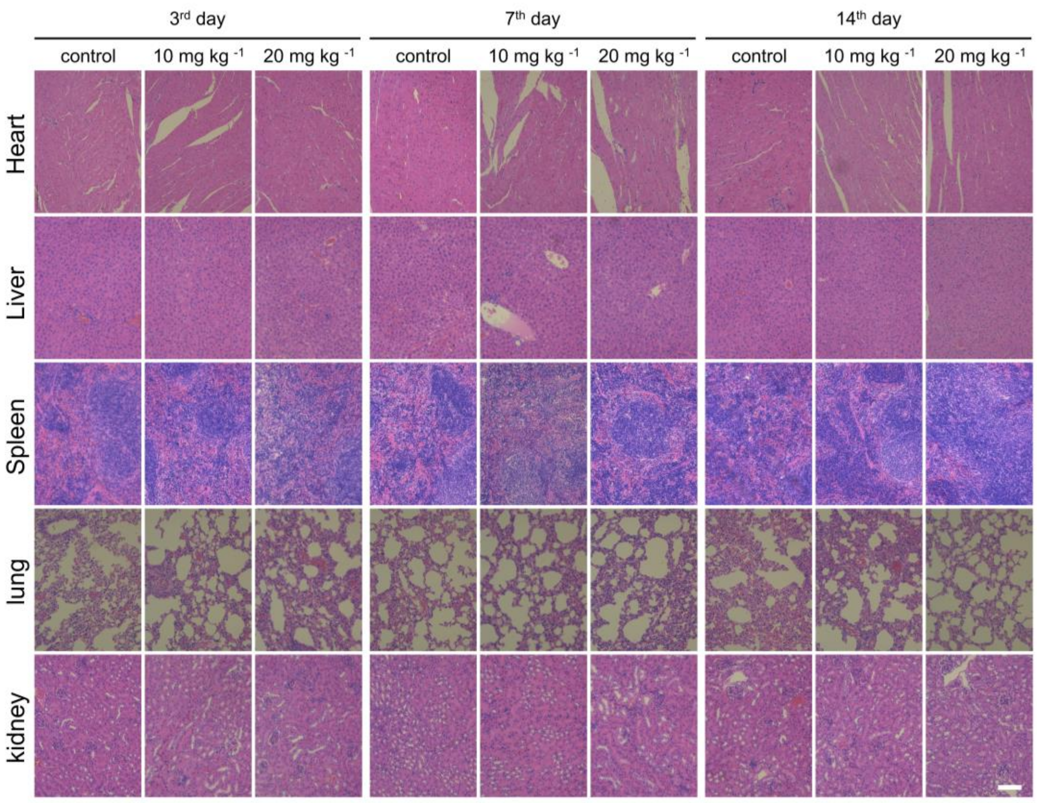

Supplementary Figure 62. Representative H&E images of main organs including the heart, liver, spleen, lung, and kidney of mice subcutaneously injected by CuN_3-SAzyme. Experiments were performed three times with similar results. Representative images are presented. Scale bar: 50 μm.

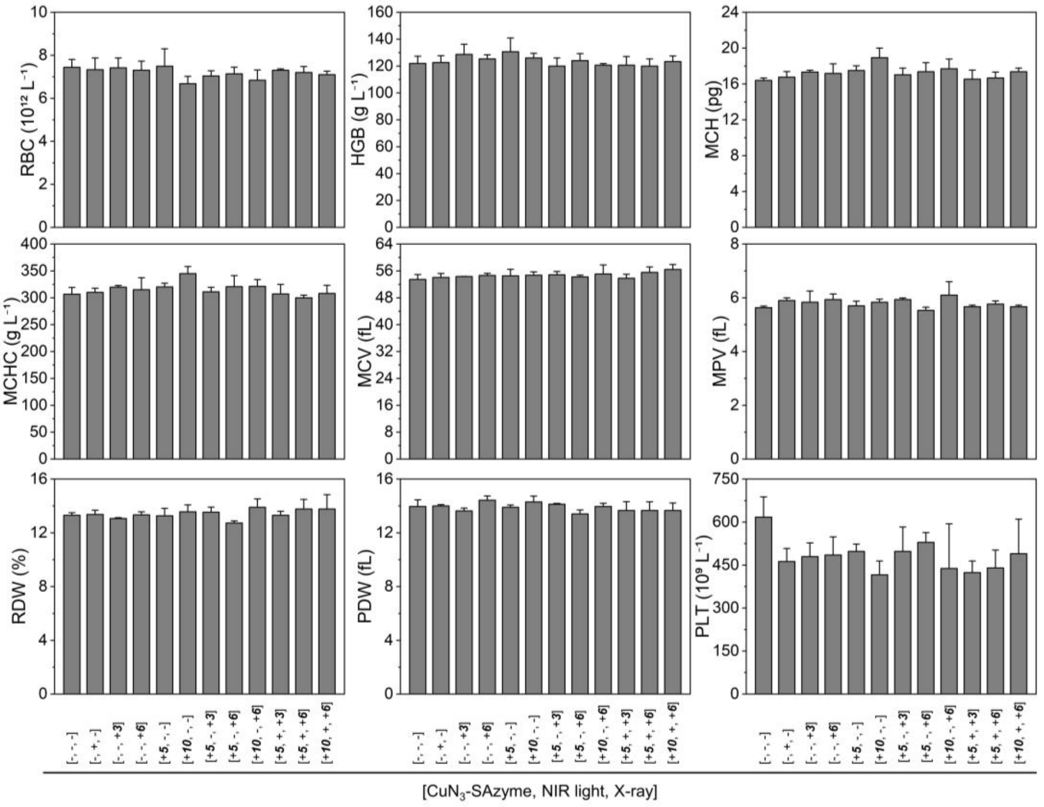

Supplementary Figure 63. Blood routine analysis of 4T1-bearing mice at the end of treatment period. All data are presented as means ± SD (n = 3 independent experiments).

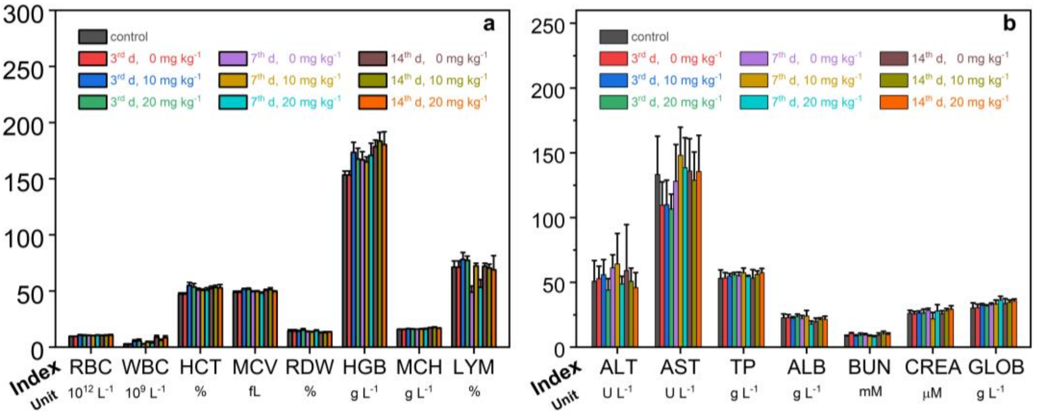

Supplementary Figure 64. Blood routine test indexes (**a**) and blood biochemistry test indicators (**b**) of mice subcutaneously injected by CuN_3-SAzyme. All data are presented as means ± SD (n = 6 biologically independent animals).

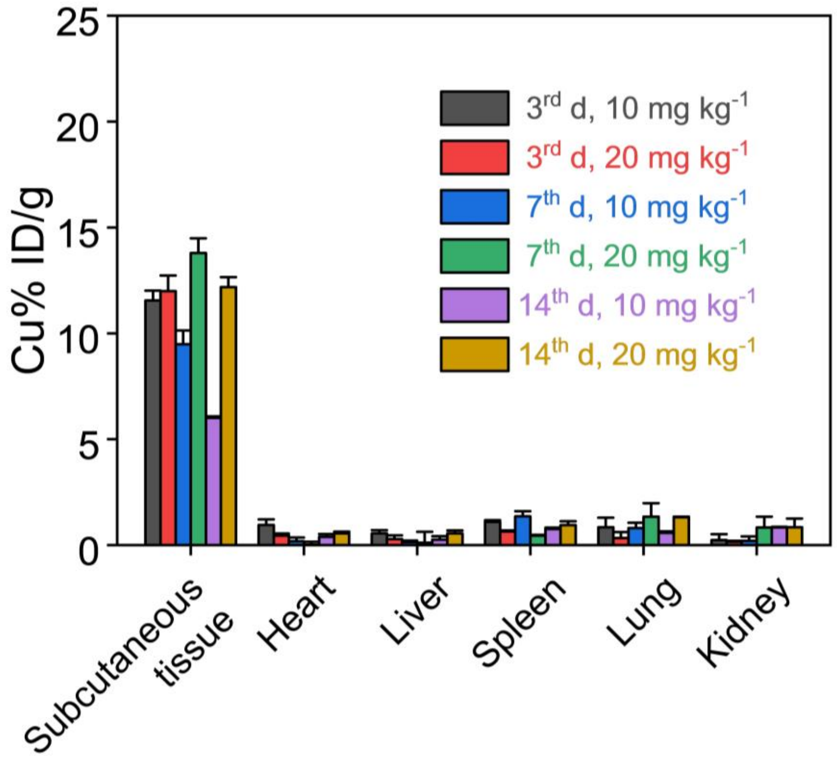

Supplementary Figure 65. Biodistribution of CuN_3-SAzyme subcutaneously injected. All data are presented as means ± SD (n = 6 biologically independent animals).

Supplementary Table 1. EXAFS fitting parameters at the Cu K-edge for various samples.

Sample	Shell	*CN*[a]	R (Å)[b]	σ^2 (10-3Å^2)[c]	ΔE_0 (eV)[d]	R factor
Cu foil	Cu-Cu	12*	2.54±0.01	8.62	4.5	0.0040
Cu_2O	Cu-O1	1.6±0.2	1.84±0.01	2.34	8.1	0.0105
	Cu-Cu	4.5±1.0	3.00±0.01	13.43		
	Cu-O2	4.5±1.0	3.48±0.01	5.93		
CuO	Cu-O1	2.5±0.3	1.95±0.01	3.30	8.9	0.0110
	Cu-O2	2.6±0.4	2.87±0.01	2.50		
CuN_4-SAzyme	Cu-N	4.2±0.5	1.98±0.01	3.26	0.6	0.0181
CuN_3-SAzyme	Cu-N	2.9±0.2	1.94±0.01	6.12	1.1	0.0041

[a]*CN*, coordination number; [b]*R*, distance between absorber and backscatter atoms; [c]σ^2, Debye-Waller factor to account for both thermal and structural disorders; [d]ΔE_0, inner potential correction; *R* factor indicates the goodness of the fit. S_0^2 was fixed to 0.86. A reasonable range of EXAFS fitting parameters: $0.600 < S_0^2 < 1.000$; $CN > 0$; $\sigma^2 > 0$ Å^2; $|\Delta E_0| < 10$ eV; R factor < 0.02.

Supplementary Table 2. Comparison of the kinetic based on Cu active sites doped on CuN_x-SAzymes and CuO_x nanozymes, for TMB substrate.

Nanozyme	pH	[E/Cu] (M)	K_M (M)	V_{max} (M min^{-1})	k_{cat} (min^{-1})	k_{cat}/K_M (M^{-1} min^{-1})
CuN_3-SAzyme	3.54	9.31×10^{-6}	1.61×10^{-3}	9.84×10^{-5}	10.57	6.56×10^{3}
CuN_4-SAzyme	3.54	3.78×10^{-6}	1.42×10^{-2}	7.80×10^{-6}	2.06	1.46×10^{2}
CuO nanozyme	3.54	3.09×10^{-4}	2.11×10^{-3}	2.04×10^{-5}	6.60×10^{-2}	31.30
Cu_2O nanozyme	3.54	3.68×10^{-4}	7.53×10^{-3}	5.34×10^{-5}	0.15	19.30

Supplementary Table 3. Comparison of the kinetic based on Cu active sites doped on CuN_x-SAzymes and CuO_x nanozymes, for H_2O_2 substrate.

Nanozyme	pH	[E/Cu] (M)	K_M (M)	V_{max} (M min^{-1})	k_{cat} (min^{-1})	k_{cat}/K_M (M^{-1} min^{-1})
CuN_3-SAzyme	3.54	9.31×10^{-6}	6.58×10^{-2}	8.76×10^{-5}	9.41	1.43×10^{2}
CuN_3-SAzyme	6.49	9.31×10^{-6}	1.94×10^{-1}	5.58×10^{-5}	5.99	30.87
CuN_4-SAzyme	3.54	3.78×10^{-6}	7.52×10^{-1}	4.80×10^{-6}	1.27	1.69
CuN_4-SAzyme	6.49	3.78×10^{-6}	3.89×10^{-1}	5.88×10^{-7}	0.16	0.40
CuO nanozyme	3.54	3.09×10^{-4}	5.30×10^{-2}	1.32×10^{-5}	4.27×10^{-2}	0.81
Cu_2O nanozyme	3.54	3.68×10^{-4}	1.13×10^{-1}	2.16×10^{-5}	5.88×10^{-2}	0.52

Supplementary Table 4. Comparison of the kinetic of CuN_3-SAzyme with various radiation treatments.

Nanozyme	pH	[E/Cu] (M)	K_M (M)	V_{max} (M min^{-1})	k_{cat} (min^{-1})	k_{cat}/K_M (M^{-1} min^{-1})	Note
CuN_3-SAzyme	3.54	2.39×10^{-7}	7.69×10^{-2}	1.66×10^{-4}	17.85	2.32×10^{2}	Under NIR light illumination
CuN_3-SAzyme	6.49	2.39×10^{-7}	2.02×10^{-1}	7.56×10^{-5}	8.12	40.14	Under NIR light illumination
CuN_3-SAzyme	3.54	2.39×10^{-7}	7.34×10^{-2}	1.41×10^{-4}	15.14	2.06×10^{2}	Under X-ray irradiation
CuN_3-SAzyme	6.49	2.39×10^{-7}	2.23×10^{-1}	6.78×10^{-5}	7.28	32.69	Under X-ray irradiation
CuN_3-SAzyme	3.54	2.39×10^{-7}	6.79×10^{-2}	8.70×10^{-5}	9.34	1.38×10^{2}	After irradiation with the dose of 20 Gy
CuN_3-SAzyme	6.49	2.39×10^{-7}	1.96×10^{-1}	5.52×10^{-5}	5.93	30.26	After irradiation with the dose of 20 Gy
CuN_3-SAzyme	3.54	2.39×10^{-7}	6.34×10^{-2}	8.64×10^{-5}	9.28	1.46×10^{2}	After irradiation with the dose of 100 Gy
CuN_3-SAzyme	6.49	2.39×10^{-7}	1.48×10^{-1}	5.04×10^{-5}	5.41	36.59	After irradiation with the dose of 100 Gy

Supplementary Table 5. Test results for submitted 4T1-Luc cells.

Loci	Test Results for Submitted Sample[a]		ExPASy Reference Database Profile		
	Query Profile: 4T1-Luc		Database Profile: 4T1		
**TH01(Human)**	-		-		
**4-2**	21.3		21.3		
**6-4**	18		18		
**1-1**	15	16	15	16	
**6-7**	12		12		
**2-1**	16	17	16	17	
**17-2**	15		15		
**11-2**	16	18	16	18	*20*
**8-1**	13		13		
**19-2**	13		13		
**7-1**	25.2		25.2		
**1-2**	17		17		
**13-1**	16.2		16.2		
**5-5**	14		14		
**12-1**	16		16		
**18-3**	18		18	*19*	
**15-3**	22.3		22.3		
**3-2**	14	15	14	15	
**X-1**	25		25		

[a]4T1-Luc cells were authenticated using Short Tandem Repeat (STR) analysis as reported by Almeida JL et al. (PLoS One 2019, 14, e0218412) The submitted profile is similar to the following ExPASy cell line: 4T1 (95.65% match). This scientific justification demonstrated that 4T1-Luc cells are not cross-contaminated or otherwise misidentified cell lines.
