# Supplementary material for: Enhancing radiation-resistance and peroxidase-like activity of single-atom copper nanozyme via local coordination manipulation
Source: Nat Commun. 2024 Jul 22;15:6174. doi: 10.1038/s41467-024-50416-8 (PMC11263674; doi:10.1038/s41467-024-50416-8)
Supplement: Supplementary file 2 — Reporting Summary [file 41467_2024_50416_MOESM2_ESM.pdf]

nature portfolio

Corresponding author(s): Liang YAN

Last updated by author(s): Jan 20, 2024

Reporting Summary

Nature Portfolio wishes to improve the reproducibility of the work that we publish. This form provides structure for consistency and transparency in reporting. For further information on Nature Portfolio policies, see our Editorial Policies and the Editorial Policy Checklist.

Statistics

For all statistical analyses, confirm that the following items are present in the figure legend, table legend, main text, or Methods section.

n/a	Confirmed	
☐	☒	The exact sample size (*n*) for each experimental group/condition, given as a discrete number and unit of measurement
☐	☒	A statement on whether measurements were taken from distinct samples or whether the same sample was measured repeatedly
☐	☒	The statistical test(s) used AND whether they are one- or two-sided<br>*Only common tests should be described solely by name; describe more complex techniques in the Methods section.*
☒	☐	A description of all covariates tested
☒	☐	A description of any assumptions or corrections, such as tests of normality and adjustment for multiple comparisons
☐	☒	A full description of the statistical parameters including central tendency (e.g. means) or other basic estimates (e.g. regression coefficient) AND variation (e.g. standard deviation) or associated estimates of uncertainty (e.g. confidence intervals)
☒	☐	For null hypothesis testing, the test statistic (e.g. *F*, *t*, *r*) with confidence intervals, effect sizes, degrees of freedom and *P* value noted<br>*Give P values as exact values whenever suitable.*
☒	☐	For Bayesian analysis, information on the choice of priors and Markov chain Monte Carlo settings
☒	☐	For hierarchical and complex designs, identification of the appropriate level for tests and full reporting of outcomes
☒	☐	Estimates of effect sizes (e.g. Cohen's *d*, Pearson's *r*), indicating how they were calculated

Our web collection on statistics for biologists contains articles on many of the points above.

Software and code

Policy information about availability of computer code

Data collection

Transmission electron microscopy images and elemental mapping were obtained from a Tecnai G2 F20 microscope operated at 200 kV (FEI, USA) and equipped with an energy dispersive X-ray analysis system.
High-angle annular dark-field scanning transmission electron microscopy (HAADF-STEM) and electron energy loss spectroscopy were performed on a JEOL-ARM300F microscope equipped with a spherical aberration corrector at 200 keV.
X-ray photoelectron spectroscopy analysis was performed by using a VG Multilab 2000 instrument (Thermo Fisher).
X-ray diffraction analysis was carried out using a D8 ADVANCE apparatus (Bruker, Germany).
Circular dichroism spectra were obtained from BioTools ChiralIR-2X.
Inductively coupled plasma optical emission spectroscopy was conducted using an Agilent 720ES apparatus.
X-ray absorption fine structure spectra at Cu K-edge were acquired at the 1W1B station in Beijing Synchrotron Radiation Facility (BSRF) and the BL11B station in Shanghai Synchrotron Radiation Facility (SSRF). All XAFS spectra were collected in ambient conditions.
Infrared thermal images were recorded by the thermal camera (FLIR Therma CAM E40).
The absorbance of ox-TMB using a microplate spectrophotometer (Multiskan MK3, Thermo Fisher Scientific, USA).
The concentration of generated oxygen was detected by a specific oxygen electrode (JPSJ-605F, INESA).
The cytotoxicity was determined via recording the absorbance at 450 nm using the microplate spectrophotometer (SpectraMax M2MDC, USA).
The live/dead cell staining fluorescent images and histopathology images were collected using an inverted fluorescence microscope (Olympus X73, Tokyo, Japan).
The intracellular reactive oxygen species, intracellular mitochondrial membrane potential fluorescent, intracellular DNA damage and cytochrome c images were recorded using a fluorescence confocal microscope (A1/LSM-Kit, Nikon/PicoQuant GmbH, Japan/Germany).
The target protein visualization was performed by enhanced chemiluminescence (ECL, Beyotime, P0018). The apoptosis cells were quantified

	by flow cytometry (BD Accuri C6, USA).<br>The absorbance of hemolysis assay the supernatant (545 nm) was detected using the microplate spectrophotometer (SpectraMax M2MDC, USA).<br>The photoacoustic images of tumors were collected on a multispectral optoacoustic tomography (MSOT, iThera Medical 128, Germany) system (750 nm to 900 nm).<br>Simulations were performed using the packages VASP (version 5.4.4) and CP2K (version 7.1). All structures were visualized using the program VESTA.
Data analysis	The acquired extended Cu K-edge X-ray absorption fine structure (EXAFS) data were processed according to the standard procedures using the ATHENA software packages.<br>Least-squares curve parameter fitting was performed using the ARTEMIS module of IFEFFIT software packages.<br>The flowcytometry data of cellular internalization were analyzed on FlowJo software (version 10.0.7).<br>The photoacoustic images of tumors were analyzed on a multispectral optoacoustic tomography (MSOT, iThera Medical 128, Germany) system (750 nm to 900 nm).<br>Data statistics and statistical significance calculation was conducted using Microsoft Excel 2016.<br>All results were expressed as mean±SD. Statistical analyses in all figures were performed by two-tailed Student's t-test. The significance level is *p < 0.05, **p < 0.01, and ***p < 0.001. Graphing software was conducted using Origin 2018.

For manuscripts utilizing custom algorithms or software that are central to the research but not yet described in published literature, software must be made available to editors and reviewers. We strongly encourage code deposition in a community repository (e.g. GitHub). See the Nature Portfolio guidelines for submitting code & software for further information.

Data

Policy information about availability of data

All manuscripts must include a data availability statement. This statement should provide the following information, where applicable:

- Accession codes, unique identifiers, or web links for publicly available datasets
- A description of any restrictions on data availability
- For clinical datasets or third party data, please ensure that the statement adheres to our policy

All data generated that support the findings of this study are present in the main text and the Supplementary Information file. Source data are provided with this paper.

Research involving human participants, their data, or biological material

Policy information about studies with human participants or human data. See also policy information about sex, gender (identity/presentation), and sexual orientation and race, ethnicity and racism.

Reporting on sex and gender	N/A
Reporting on race, ethnicity, or other socially relevant groupings	N/A
Population characteristics	N/A
Recruitment	N/A
Ethics oversight	N/A

Note that full information on the approval of the study protocol must also be provided in the manuscript.

Field-specific reporting

Please select the one below that is the best fit for your research. If you are not sure, read the appropriate sections before making your selection.

☒ Life sciences ☐ Behavioural & social sciences ☐ Ecological, evolutionary & environmental sciences

For a reference copy of the document with all sections, see nature.com/documents/nr-reporting-summary-flat.pdf

Life sciences study design

All studies must disclose on these points even when the disclosure is negative.

Sample size	No statistical methods were used to predetermine the sample sizes. The specific sample sizes in the experiments have been indicated in the manuscript. The sample size (usually n≥3 biologically independent samples) was determined by allowable error, accuracy and resources.<br>The sample sizes of in vivo experiments (at least three animals in each treatment group) represents the minimum number of animals needed to reach statistical significance (p<0.05) between experimental groups. The sample sizes are consistent with those generally adopted and accepted in this field.<br>1. W. Feng, et al. 2D vanadium carbide MXenzyme to alleviate ROS-mediated inflammatory and neurodegenerative diseases. Nature Commun. 2021, 12(1): 2203.

	2. S. Ji, et al. Matching the kinetics of natural enzymes with a single-atom iron nanozyme. Nature Catal. 2021, 4(5): 407-417.
Data exclusions	No data were excluded from the analyses.
Replication	For catalytic kinetic experiments, individual experiment was independently repeated 3 times. For in vitro experiments, 6 biologically independent samples were used. For in vivo experiments, the displayed data collected from 5/6 biologically independent animals. Every experiment included replicates as described in the Figure legends.
Randomization	All experimental samples and animal models were allocated randomly to each group.
Blinding	In the animal experiments, the main investigators were blinded in the process of group allocation. In other experiments, the main investigators were not blinded since the experimental design, execution and data analysis were performed by the same person.

Reporting for specific materials, systems and methods

We require information from authors about some types of materials, experimental systems and methods used in many studies. Here, indicate whether each material, system or method listed is relevant to your study. If you are not sure if a list item applies to your research, read the appropriate section before selecting a response.

Materials & experimental systems

n/a	Involved in the study
☐	☒ Antibodies
☐	☒ Eukaryotic cell lines
☒	☐ Palaeontology and archaeology
☐	☒ Animals and other organisms
☒	☐ Clinical data
☒	☐ Dual use research of concern
☒	☐ Plants

Methods

n/a	Involved in the study
☒	☐ ChIP-seq
☐	☒ Flow cytometry
☒	☐ MRI-based neuroimaging

Antibodies

Antibodies used

1. ki-67 Rabbit Monoclonal Antibody (Beyotime, Cat# AF1738, 1:200 dilution)
2. Phospho-Histone H2A.X (Ser139) Mouse Monoclonal Antibody (Beyotime, Cat# AG2114 1:1000 dilution)
3. Cy3-conjugated sheep anti-mouse secondary antibody (Beyotime, Cat# A0521, 1:1000 dilution)
4. Cytochrome C antibody (Beyotime, Cat# AC908, 1:1000 dilution)
5. Alexa Fluor 488-labeled sheep anti-mouse secondary antibody (Beyotime, Cat# A0428, 1:1000 dilution)
6. β-Actin Mouse Monoclonal Antibody (Beyotime, Cat# AF0003, 1:1000 dilution)
7. HRP-labeled sheep anti-mouse secondary antibody (Beyotime, Cat# A0216, 1:1000 dilution)
8. HSP70 Mouse Monoclonal Antibody (Beyotime, Cat# AG2202, 1:1000 dilution)
9. Cleaved Caspase-3 (Asp175) Antibody(Cell Signaling Technology, #9661, 1:1000 dilution)

Validation

All antibodies were commercially available and were validated by the supplier. All antibodies were used in the study according to the profile of manufacturers. Validation statements are provided on the manufacturer's website.

1. ki-67 Rabbit Monoclonal Antibody (Beyotime, Cat# AF1738, 1:200 dilution)
https://www.beyotime.com/product/AF1738.htm
2. Phospho-Histone H2A.X (Ser139) Mouse Monoclonal Antibody (Beyotime, Cat# AG2114 1:1000 dilution)
https://www.beyotime.com/product/AG2114.htm
3. Cy3-conjugated sheep anti-mouse secondary antibody (Beyotime, Cat# A0521, 1:1000 dilution)
https://www.beyotime.com/product/A0521.htm
4. Cytochrome C antibody (Beyotime, Cat# AC908, 1:1000 dilution)
https://www.beyotime.com/product/AC908.htm
5. Alexa Fluor 488-labed sheep anti-mouse secondary antibody (Beyotime, Cat# A0428, 1:1000 dilution)
https://www.beyotime.com/product/A0428.htm
6. β-Actin Mouse Monoclonal Antibody (Beyotime, Cat# AF0003, 1:1000 dilution)
https://www.beyotime.com/goods.do?method=search&q=AF0003
7. HRP-labeled sheep anti-mouse secondary antibody (Beyotime, Cat# A0216, 1:1000 dilution)
https://www.beyotime.com/product/A0216.htm
8. HSP70 Mouse Monoclonal Antibody (Beyotime, Cat# AG2202, 1:1000 dilution)
https://www.beyotime.com/product/AG2202.htm
9. Cleaved Caspase-3 (Asp175) Antibody (Cell Signaling Technology, #9661, 1:1000 dilution)
https://www.cellsignal.com/products/primary-antibodies/cleaved-caspase-3-asp175-antibody/9661

Eukaryotic cell lines

Policy information about cell lines and Sex and Gender in Research

Cell line source(s)	4T1-Luc, 3T3, K7M2 cell lines were purchased from Peking Union Medical College Hospital.
Authentication	American Type Culture Collection and Hunan Fenghui Biotechnology Co., Ltd used morphology, karyotyping, and PCR based approaches to confirm the identity of cell lines and to rule out both intra- and interspeies contamination. Also, the cell lines were frequently checked by their morphological features.
Mycoplasma contamination	All cells were negative for mycoplasma.
Commonly misidentified lines (See ICLAC register)	No commonly misidentified cell line were used.

Animals and other research organisms

Policy information about studies involving animals; ARRIVE guidelines recommended for reporting animal research, and Sex and Gender in Research

Laboratory animals	BALB/c mice (4-6 weeks, female; 6-8 weeks old, female) and 4T1-bearing BALB/c nude mice (5-6 weeks old, male) were provided by Beijing HFK Bioscience Co., Ltd. All mice were housed in SPF-grade facilities, cages with standard conditions (50% relative humidity and 12/12 h light-dark cycle) at 25 °C.
Wild animals	No wild animals were used in this study.
Reporting on sex	The present study involves female study design, experiments and results.
Field-collected samples	This study did not involve samples collected from the fields.
Ethics oversight	All animal experiments were performed according to the published guidelines of the CAS Key Laboratory for Biomedical Effects of Nanomaterials and Nanosafety, Institute of High Energy Physics and National Center for Nanoscience and Technology (Approval ID: IHEPLLSC-2023-52). All mice were kept in accordance with the ethics committee of the National Ministry of Health.

Note that full information on the approval of the study protocol must also be provided in the manuscript.

Plants

Seed stocks	*Report on the source of all seed stocks or other plant material used. If applicable, state the seed stock centre and catalogue number. If plant specimens were collected from the field, describe the collection location, date and sampling procedures.*
Novel plant genotypes	*Describe the methods by which all novel plant genotypes were produced. This includes those generated by transgenic approaches, gene editing, chemical/radiation-based mutagenesis and hybridization. For transgenic lines, describe the transformation method, the number of independent lines analyzed and the generation upon which experiments were performed. For gene-edited lines, describe the editor used, the endogenous sequence targeted for editing, the targeting guide RNA sequence (if applicable) and how the editor was applied.*
Authentication	*Describe any authentication procedures for each seed stock used or novel genotype generated. Describe any experiments used to assess the effect of a mutation and, where applicable, how potential secondary effects (e.g. second site T-DNA insertions, mosiacism, off-target gene editing) were examined.*

Flow Cytometry

Plots

Confirm that:

- [x] The axis labels state the marker and fluorochrome used (e.g. CD4-FITC).
- [x] The axis scales are clearly visible. Include numbers along axes only for bottom left plot of group (a 'group' is an analysis of identical markers).
- [x] All plots are contour plots with outliers or pseudocolor plots.
- [x] A numerical value for number of cells or percentage (with statistics) is provided.

Methodology

Sample preparation	Cells were stained by annexin V-FITC (AV, Dojindo, Japan) and propidium iodide (PI, Dojindo, Japan) for 15 min at 37 ºC in the dark. Finally, the apoptosis cells were quantified by flow cytometry (BD Accuri C6, USA) in FITC and/or PI channel
Instrument	BD Accuri™ C6 Plus Personal Flow Cytometer.

Software	The flowcytometry data were analyzed on FlowJo software (version 10.0.7).
Cell population abundance	No sorting was performed.
Gating strategy	Generally, cells were first gated on FSC/SSC. Singlet cells were gated using FSC-H and FSC-A.

☒ Tick this box to confirm that a figure exemplifying the gating strategy is provided in the Supplementary Information.
